# Roles of ACSL4/GPX4 and FSP1 in oxalate-induced acute kidney injury

**DOI:** 10.1038/s41420-025-02557-y

**Published:** 2025-06-17

**Authors:** Keng Ye, Ruilong Lan, Zhimin Chen, Kunmei Lai, Yankun Song, Guoping Li, Huabin Ma, Hong Chen, Yanfang Xu

**Affiliations:** 1 Department of Nephrology, Blood Purification Research Center, the First Affiliated Hospital, Fujian Medical University, Fuzhou, 350005 China; 2 Research Center for Metabolic Chronic Kidney Disease, the First Affiliated Hospital, Fujian Medical University, Fuzhou, 350005 China; 3 Department of Nephrology, National Regional Medical Center, Binhai Campus of the First Affiliated Hospital, Fujian Medical University, Fuzhou, 350212 China; 4 Central Laboratory, the First Affiliated Hospital, Fujian Medical University, Fuzhou, 350005 China; 5 Department of Pathology, the First Affiliated Hospital, Fujian Medical University, Fuzhou, 350005 China

**Keywords:** Cell death, Acute kidney injury

## Abstract

Ferroptosis has emerged as a crucial driver of injury in various organs, including acute kidney injury (AKI). However, the regulatory roles and underlying mechanisms of key genes involved in ferroptosis during oxalate-induced AKI are not fully understood. In this study, we conducted single-cell RNA sequencing (scRNA-seq) analysis of kidney samples, revealing the occurrence of ferroptosis in renal tubular cells of an oxalate-induced AKI mouse model, which was confirmed in subsequent in vitro experiments. Furthermore, renal tubule-specific deficiency of *Acsl4* conferred significant protection against oxalate-induced AKI, as evidenced by alleviated structural and functional renal damage, reduced oxidative stress and decreased inflammatory cell infiltration, all of which collectively contribute to a reduction in ferroptosis. In contrast, *Fsp1* deficiency exacerbated these pathological processes. Consistent with the in vivo findings, *Acsl4* knockout in mouse renal tubular epithelial cell lines (MTECs) resulted in decreased lipid peroxidation and mitigation of mitochondrial dysfunction, thus reducing calcium oxalate (CaOX)-induced ferroptosis. Conversely, *Fsp1* knockout in MTECs had the opposite effects. In addition, as expected, overexpression of the ferroptosis inhibitors GPX4 or FSP1 in MTECs significantly reduced CaOX-induced lipid peroxidation and cell ferroptosis. In summary, these findings indicated that oxalate exposure upregulated ferroptosis driver ACSL4 and downregulated inhibitors like GPX4 and FSP1, leading to lipid peroxidation and mitochondrial dysfunction, which collectively triggered ferroptosis in renal tubular cells. Modulating ACSL4/GPX4 and FSP1 axes presents a promising therapeutic strategy for oxalate-induced AKI.

## Introduction

Crystal nephropathies [1] are a group of disorders characterized by the deposition of crystals in the renal tubules or interstitium, resulting in kidney dysfunction. Crystal accumulation can cause significant damage, including tubular obstruction, oxidative stress, and inflammation, potentially leading to AKI or chronic kidney disease (CKD). Crystalline-related diseases, despite their different clinical presentations, share common pathological and molecular mechanisms, leading to their collective classification as “crystallopathies” [2]. Crystal nephropathies are classified based on the site of crystal deposition into renal vascular, tubular, and urolithiasis-related nephropathies [1], often accompanied by the formation of kidney stones. The prevalence of kidney stones is 10.6% in men and 7.1% in women, with a high recurrence rate—50% within 5 years and up to 80% within 10 years [3]. In recent years, the incidence of kidney stones has been increasing, with rates ranging from 1 to 5% in Asia, 5–9% in Europe, and 7–13% in North America [4]. Growing evidence links kidney stones with obesity, diabetes, and cardiovascular diseases [5, 6]. The risk of kidney stones is also higher in individuals with hypertension [7], bone disorders [8], and metabolic syndrome [5]. Moreover, kidney stones can lead to CKD and renal failure [9, 10]. Thus, investigating the mechanisms of crystal nephropathies and identifying therapeutic targets is crucial.

Oxalate-induced acute kidney injury (AKI) is a common form of tubular crystal nephropathy. This condition results in the formation of CaOX crystals, which gradually accumulate in the renal interstitium or collecting ducts, eventually causing nephrocalcinosis [11]. This accumulation causes significant inflammation and cell death in renal tubular epithelial cells (RTCs), ultimately resulting in renal calcification, hematuria, and, in severe cases, systemic oxalosis with progression to end-stage renal failure. Previous research has demonstrated that oxalate crystals cause ongoing damage to RTCs, triggering inflammatory responses closely linked to kidney stone formation. Reactive oxygen species (ROS)-induced oxidative stress plays a key regulatory role in this process [12], with mitochondrial damage being a major contributor to ROS production in RTCs exposed to crystals [13]. Crystal-induced necroinflammation engages multiple signaling pathways, resulting in various forms of cell death, such as apoptosis, necroptosis, and ferroptosis [14]. Therefore, targeting a single cytokine or pathway may be inadequate to alleviate kidney injury.

Ferroptosis, a term introduced in 2012 [15], is a form of regulated cell death that relies on iron and is driven by excessive lipid peroxidation [16]. Several key regulatory factors are involved in the process of ferroptosis. For instance, ACSL4 promotes the incorporation of PUFAs into phospholipids, making them vulnerable to lipoxygenase (ALOX)-mediated oxidation, which compromises the lipid bilayer and induces ferroptosis [17, 18]. In contrast, GPX4 prevents ferroptosis by using GSH to convert lipid hydroperoxides into non-toxic lipid alcohols, reducing oxidative stress and protecting against ferroptosis [19]. Additionally, FSP1 functions independently of glutathione, producing metabolites that trap free radicals, thereby suppressing ferroptosis [20, 21]. Over the past few years, ferroptosis has been reported to be associated with the development and treatment responses of various tumors, as well as neurodegenerative diseases, ischemic organ injuries and kidney disease [22–24]. Despite the unclear regulatory mechanisms of ferroptosis, particularly in the initiation and progression of various diseases, it is still widely regarded as a promising target for developing new treatment strategies [16].

Ferroptosis contributes to the development of AKI. For instance, Gao X et al. identified the differential expression of ACSL4 in folic acid-induced AKI mice, and found that its knockout significantly reduced ferroptosis, thereby mitigating tissue damage, inflammation, and macrophage infiltration [25]. Similarly, a study by Andreas Linkermann and colleagues demonstrated that mice with *Fsp1* deficiency or GPX4 dysfunction exhibit increased sensitivity of renal tubular epithelial cells to ferroptosis during AKI [26]. Despite numerous related studies, it remains uncertain whether these lipid metabolism-related axes could provide a solid basis for developing effective therapeutic strategies for oxalate-induced AKI. In this study, we thoroughly investigated ACSL4/GPX4 and FSP1-mediated ferroptosis in oxalate-induced AKI, highlighting that targeting ferroptosis in renal tubular epithelial cells may be a promising strategy for treating and preventing the progression of this oxalate-induced nephropathy.

## Results

### Renal tubular cell ferroptosis was induced in kidney after oxalate treatment

**Fig. 1:**
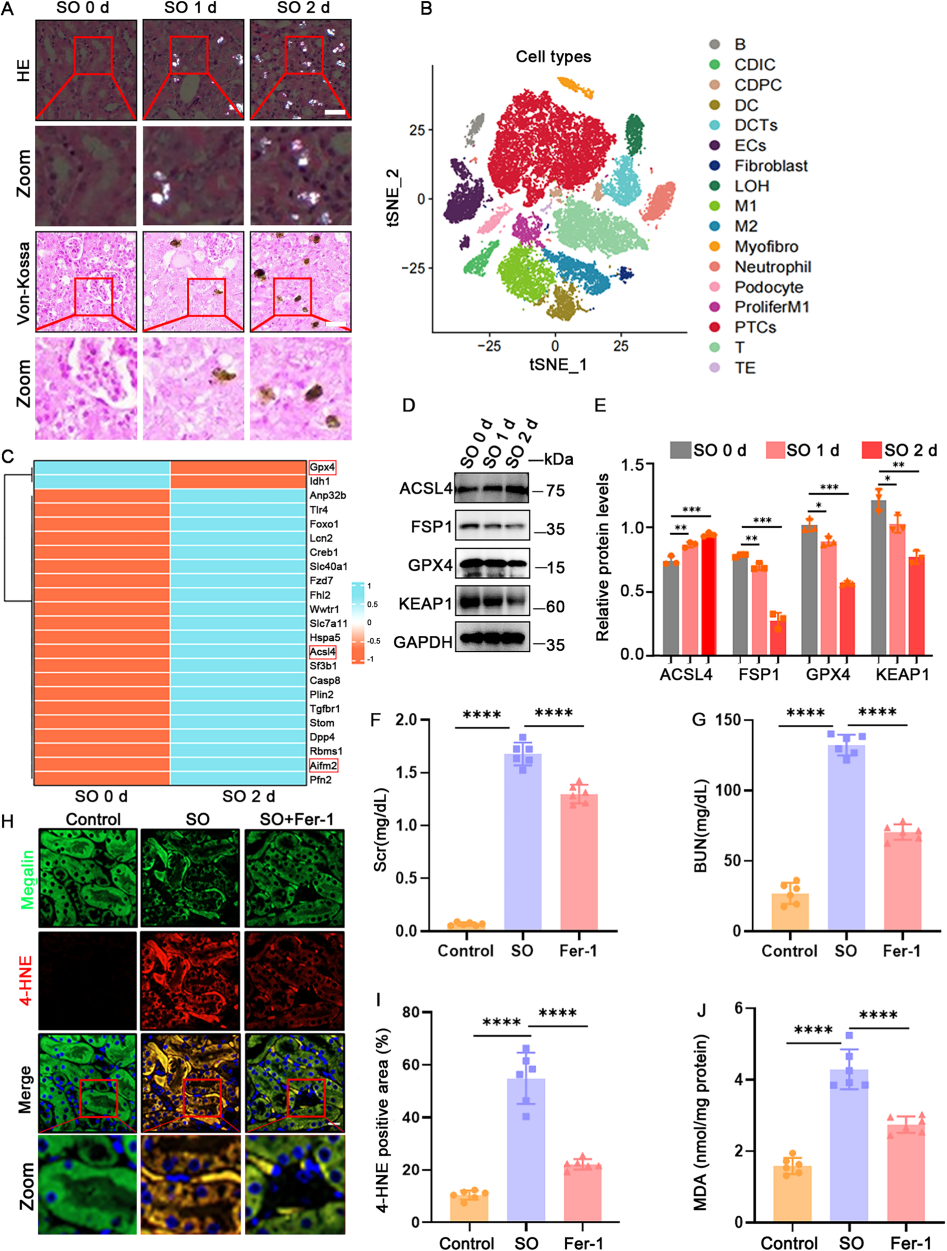
Ferroptosis was triggered in renal tubular cells in oxalate-induced AKI. **A** Representative images of HE staining showing crystal deposition in mouse kidney tissue, and Von-Kossa staining for black CaOX crystal deposits (*n* = 6, scale bar = 30 μm). The SO 1d and SO 2d groups represent mice injected with NaOX (100 mg/kg) and provided 3% NaOX in their drinking water for 1 or 2 days to induce oxalate-induced AKI, while the SO 0d group received an equivalent volume of saline injection and regular drinking water without NaOX. **B** tSNE plots derived from scRNA-seq analysis showing cell clusters identified in the kidneys of the oxalate-induced AKI mouse model (*n* = 3). **C** The heatmap showing altered expression of ferroptosis-related genes in the kidneys of the mouse model, with upregulated or downregulated genes in orange or blue, respectively (*n* = 3). **D**, **E** Western blot and quantitative analysis for further confirmation of ferroptosis-related protein levels in purified renal proximal tubule tissues from the indicated groups (*n* = 3). **F**, **G** Measurement of Scr and BUN levels in the indicated groups of mice. **H**, **I** Immunofluorescence staining and corresponding quantitative analysis of 4-HNE (red), Megalin (green), and DAPI (blue) in kidneys injected intraperitoneally with 100 mg/kg NaOX for 2 days, administering 5 mg/kg Fer-1 (*n* = 6). **J** Quantification of MDA content of the kidney at 2 days after intraperitoneal injection with 100 mg/kg NaOX (*n* = 6). ns nonsignificant, *p < 0.05, **p < 0.01, ***p < 0.001, ****p < 0.0001.

Herein, we developed a mouse model of oxalate-induced AKI. HE and Von-Kossa staining showed that kidneys exhibited tubular damage, inflammatory infiltration, and time-dependent CaOX crystal deposition (Fig. 1A), confirming the successful establishment of the model.

To evaluate the renal transcriptome profile in the oxalate-induced AKI mouse model, kidney samples were subjected to 10× scRNA-seq. The analysis identified 17 cell subpopulations, with the proximal tubular cells (PTCs) being the most enriched (Fig. 1B). The heatmap revealed significant alterations in the expression of ferroptosis-related genes, including *Acsl4, Gpx4*, and *Aifm2* (*Fsp1*) after NaOX treatment (Fig. 1C), suggesting a critical role of ferroptosis in oxalate-induced AKI.

To validate the occurrence of ferroptosis in the mouse model, proteins from renal proximal tubular tissues were analyzed using Western blot. The results showed a significant upregulation of ACSL4, while FSP1, GPX4, and KEAP1 were notably downregulated in the NaOX administration groups (Fig. 1D, E and Supplementary uncropped Western blots Fig. 1D). Oxalate-induced AKI mice exhibited significantly elevated levels of blood urea nitrogen (BUN) and serum creatinine (Scr). Notably, treatment with ferroptosis-specific inhibitor Ferrostatin-1 (Fer-1), significantly reduced BUN and Scr levels in Oxalate-induced AKI mice (Fig. 1F, G). Furthermore, administration of Fer-1 effectively rescued oxalate-induced lipid peroxidation, manifested by decreased MDA levels and reduced 4-HNE production (Fig. 1H–J). Consistently, in vitro experiments using primary PTECs treated with 6 mM CaOX for 10 or 20 h showed increased ACSL4 levels and decreased FSP1, GPX4, and KEAP1 levels (Fig. S1A, B and Supplementary uncropped Western blots Fig. S1A). To further elucidate the role of ferroptosis in PTECs following CaOX treatment, cells were co-treated with the ferroptosis-specific inhibitors Liproxstatin-1 (Lip-1) or Fer-1, resulting in significant reductions in CaOX-induced cell death (Fig. S1C, D). Western blot analysis further demonstrated that these inhibitors reversed the CaOX-induced changes in ACSL4, FSP1, GPX4, and KEAP1 (Fig. S1E and Supplementary uncropped Western blots Fig. S1E). Together, these data provided strong evidence that ferroptosis was induced in renal tubular cells in oxalate-induced AKI.

### *Acsl4* deletion attenuated renal impairment and oxidative stress in the oxalate-induced AKI mouse model

**Fig. 2:**
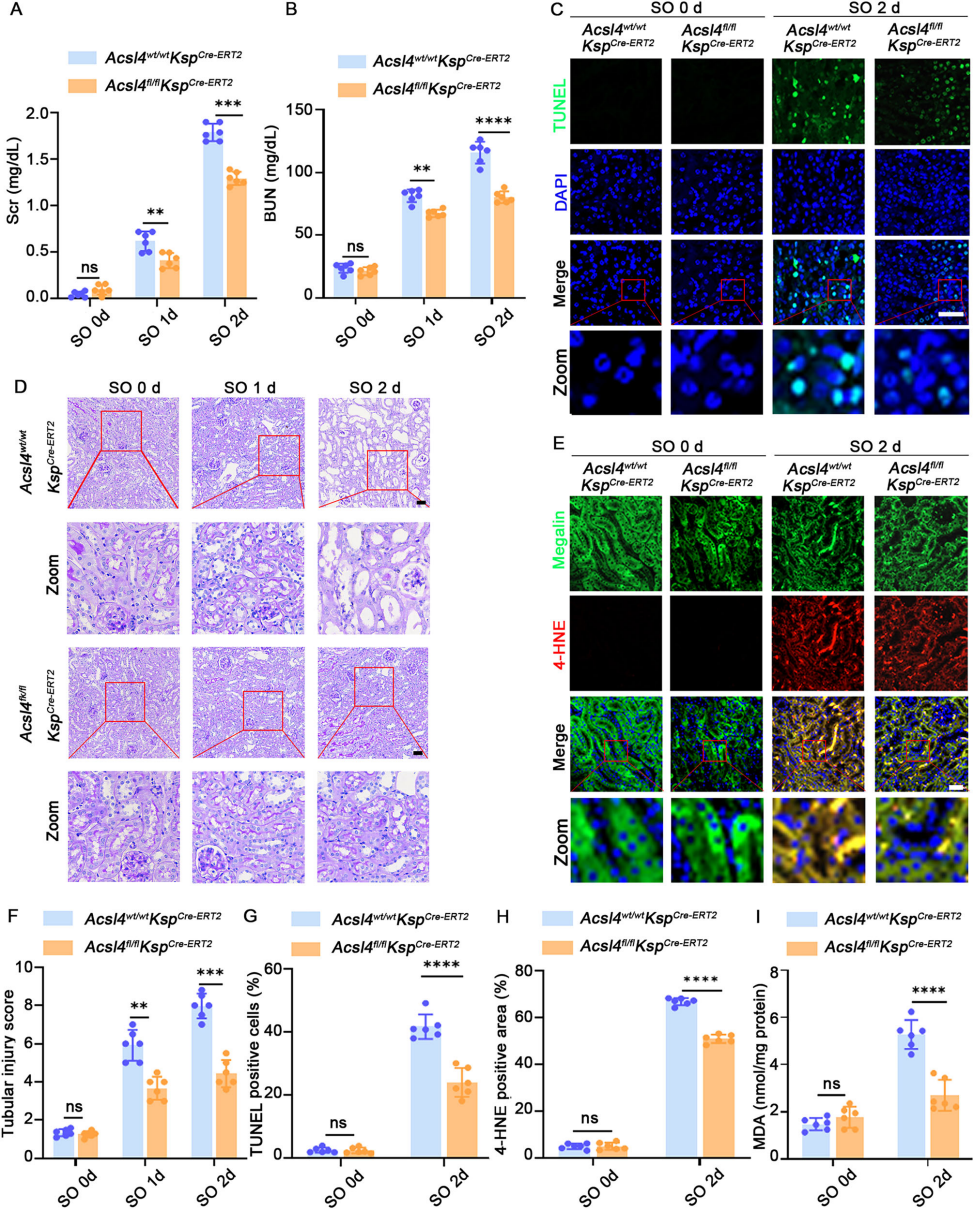
Deletion of *Acsl4* alleviated renal dysfunction and oxidative stress in the oxalate-induced AKI mouse model. **A**, **B** Evaluation of kidney function in the indicated groups of mice, including the measurement of Scr and BUN levels. **D** PAS staining of kidney sections (scale bar = 30 μm), **F** Tubular injury score, **C**, **G** representative TUNEL staining images and corresponding quantitative analysis (TUNEL: green, DAPI: blue, scale bar = 50 μm) and (**E**, **H**) 4-HNE staining and corresponding quantitative analysis (Megalin: green, 4-HNE (a sensitive marker of lipid peroxidation): red, DAPI: blue, scale bar = 50 μm) of the kidney tissues from the indicated groups. **I** Quantification of MDA content of the kidney at 2 days after intraperitoneal injection with NaOX. All *n* = 6. ns, nonsignificant, *p < 0.05, **p < 0.01, ***p < 0.001, ****p < 0.0001.

To clarify the significance of ferroptosis in oxalate-induced AKI, we first generated a mouse model with renal tubule-specific deletion of *ACSL4* (*Acsl4*^*fl/fl*^*Ksp*^*Cre-ERT2*^) (Fig. S2A, C, E). Compared to the *Acsl4*^*wt/wt*^*Ksp*^*Cre-ERT2*^ groups, the *Acsl4*^*fl/fl*^*Ksp*^*Cre-ERT2*^ groups exhibited lower Scr and BUN levels after NaOX treatment, indicating milder renal function impairment (Fig. 2A, B). Moreover, PAS staining showed that *Acsl4*^*fl/fl*^*Ksp*^*Cre-ERT2*^ mice revealed only mild tubular injury and dilation, whereas *Acsl4*^*wt/wt*^*Ksp*^*Cre-ERT2*^ mice showed evident tubular necrosis and dilation following NaOX treatment (Fig. 2D). Additionally, the *Acsl4*^*fl/fl*^*Ksp*^*Cre-ERT2*^ groups also exhibited lower tubular injury scores compared to the *Acsl4*^*wt/wt*^*Ksp*^*Cre-ERT2*^ groups (Fig. 2F). Furthermore, the *Acsl4*^*fl/fl*^*Ksp*^*Cre-ERT2*^ mice showed reduced cell death, as reflected by a lower TUNEL-positive cell ratio (Fig. 2C, G) and decreased renal oxidative stress injury, as indicated by a lower 4-HNE staining (Fig. 2E, H) and MDA levels (Fig. 2I), compared to the *Acsl4*^*wt/wt*^*Ksp*^*Cre-ERT2*^ mice after NaOX treatment. Immunofluorescence staining showed that the proportion of F4/80 and Ly6G positive cells infiltrating the renal tubular interstitium was lower in the former group compared to the latter (Fig. S3A, B), which was further confirmed by flow cytometry analysis (Fig. S3C, D). These findings suggested that renal tubule-specific deletion of *Acsl4* reduced ferroptosis occurrence and offered protection against oxalate-induced AKI.

### Loss of *Fsp1* aggravated renal dysfunction and amplified oxidative stress in oxalate-induced AKI mice

**Fig. 3:**
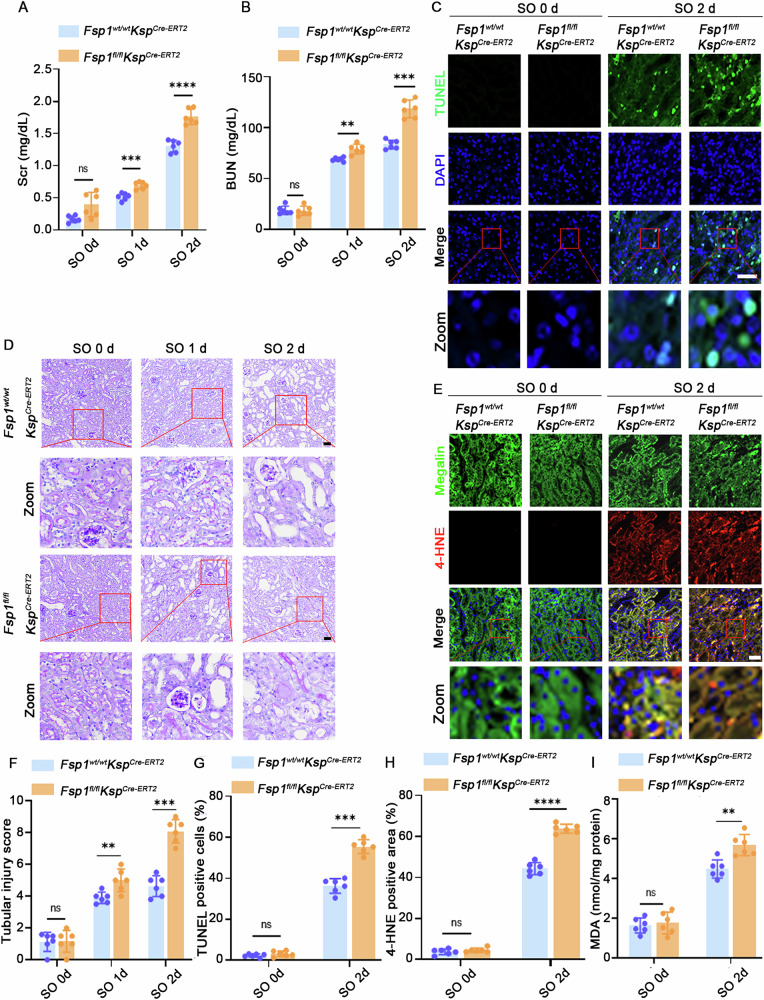
Loss of *Fsp1* exacerbated renal dysfunction and oxidative stress in oxalate-induced AKI mice. **A**, **B** Assessment of renal function in the mice in the designated group, with measurements of Scr and BUN levels. **D** PAS staining (scale bar = 30 μm), **F** tubular injury score, (**C**, **G**, **E**, **H**) TUNEL staining and 4-HNE staining and their corresponding quantitative analysis (scale bar = 50 μm) of the kidney tissues for the indicated groups. **I** Quantification of MDA content of the kidney at 2 days after intraperitoneal injection with NaOX. All *n* = 6. ns nonsignificant, *p < 0.05, **p < 0.01, ***p < 0.001.

In addition, we developed a renal tubule-specific *Fsp1* knockout mouse model *Fsp1*^*fl/fl*^*Ksp*^*Cre-ERT2*^ (Fig. S2B, C, F) to further investigate the effects of ferroptosis in oxalate-induced AKI. Our data showed that the *Fsp1*^*fl/fl*^*Ksp*^*Cre-ERT2*^ groups displayed higher Scr and BUN levels than those of the *Fsp1*^*wt/wt*^*Ksp*^*Cre-ERT2*^ groups following NaOX treatment (Fig. 3A, B), indicating more severe renal function. Furthermore, PAS staining revealed more severe renal pathology in the *Fsp1*^*fl/fl*^*Ksp*^*Cre-ERT2*^ groups, characterized by greater tubular necrosis, dilation and higher injury scores (Fig. 3D, F). Additionally, the *Fsp1*^*fl/fl*^*Ksp*^*Cre-ERT2*^ mice showed an increased TUNEL-positive cell ratio (Fig. 3C, G), a higher level of 4-HNE staining (Fig. 3E, H) and MDA levels (Fig. 3I) compared to the *Fsp1*^*wt/wt*^*Ksp*^*Cre-ERT2*^ mice after NaOX treatment. Immunofluorescence staining revealed an increased infiltration of F4/80⁺ and Ly6G⁺ cells in the renal tubular interstitium of the former group relative to the latter group (Fig. S4A, B). This observation was further validated by flow cytometry (Fig. S4C, D). These data indicated that renal tubule-specific deletion of *Fsp1* promoted ferroptosis and aggravated oxalate-induced AKI. Interestingly, the oxalate-induced AKI model was accompanied by the release of HMGB1 (Fig. S5A, B) and ferroptosis inhibitor Fer-1 effectively reduces inflammation, evidenced by decreased F4/80⁺ and Ly6G⁺ immune cell infiltration (Fig. S5C, D). These findings collectively suggested a strong correlation between ferroptosis and immune cell recruitment in oxalate-induced AKI.

### *Acsl4* knockout reduced CaOX-induced ferroptosis in MTECs

**Fig. 4:**
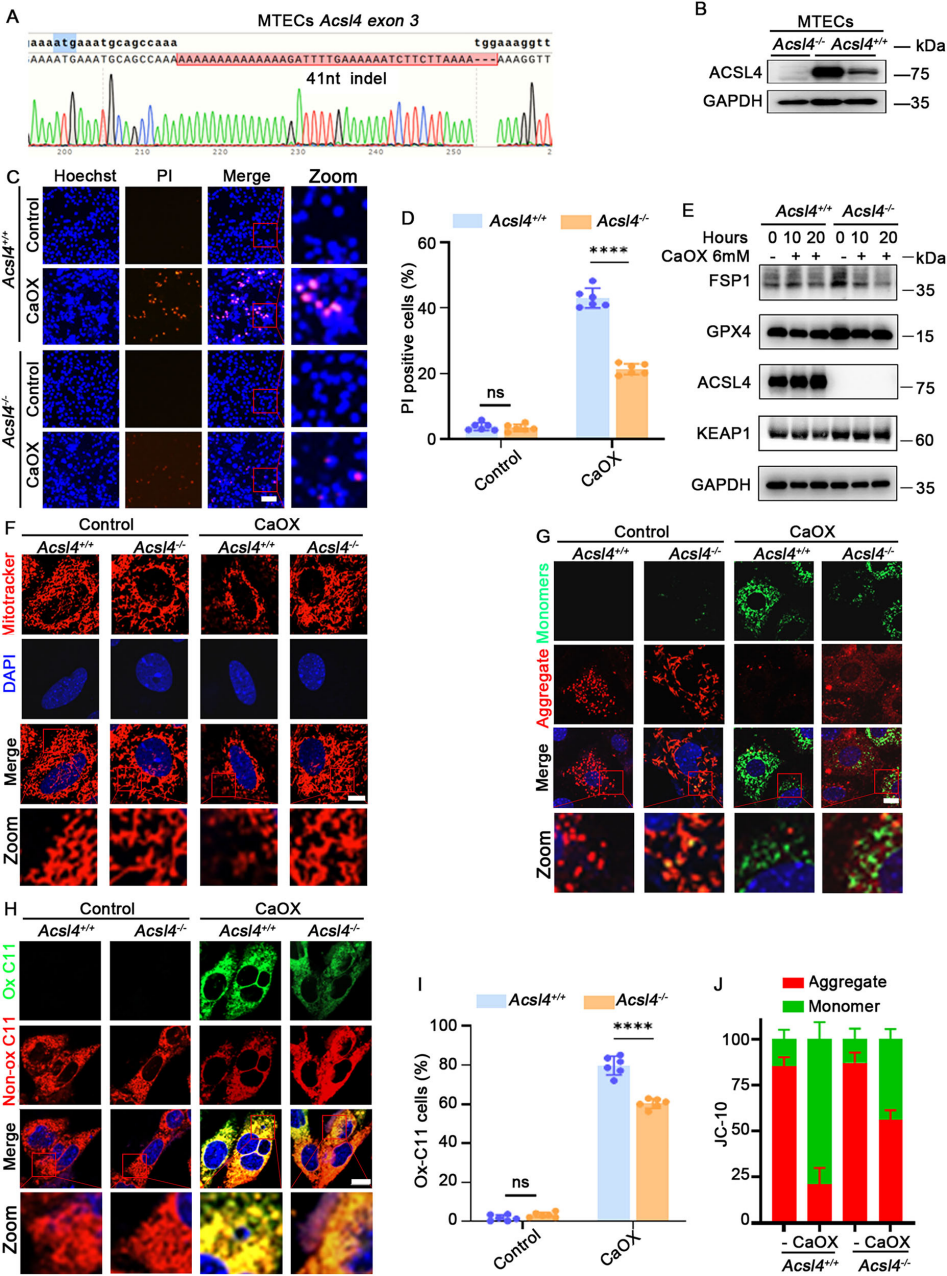
Knockout of the *Acsl4* gene reduced ferroptosis in MTECs. **A**, **B** Sanger sequencing and Western blot validation for *Acsl4* knockdown in MTECs. **C**, **D** Representative images of PI/Hoechst staining and corresponding quantitative analysis in MTECs with 6 mM CaOX treatment for 20 h (*n* = 6, scale bar = 80 μm) and (**E**) the expression of ACSL4, FSP1, GPX4, and KEAP1 in MTECs after 6 mM CaOX treatment for 10 or 20 h. **F** Mito-Tracker Red CMXRos probe staining for examining the morphology of mitochondria, **G**, **J** JC-10 staining for assessing mitochondrial membrane potential with quantitative analysis of the ratio between red aggregates and green monomers and **H**, **I** representative images of BODIPY 581/591 C11 lipid peroxidation fluorescent probe staining with quantitative analysis for the oxidized (Ox-C11) cell ratios in MTECs with 6 mM CaOX treatment for 12 h (all *n* = 6, scale bar = 10 μm). ns nonsignificant, ****p < 0.0001.

The above in vivo experiments demonstrated that ferroptosis played a critical role in oxalate-induced AKI. To further confirm its effects in this process, we modulated the expression of key regulators of ferroptosis in vitro. First, we utilized the CRISPR-Cas9 system to generate *Acsl4* knockout MTECs and confirmed the knockout efficiency via Sanger sequencing and Western blot analysis (Fig. 4A, B and Supplementary uncropped Western blots Fig. 4B). Notably, cells treated with CaOX showed a reduced PI-positive ratio in the *Acsl4* knockout (*Acsl4*^*−/−*^) group compared to the *Acsl4* wild-type (*Acsl4*^*+/+*^) group (Fig. 4C, D). Additionally, increased expression of GPX4 and KEAP1 was observed in *Acsl4*^*-/-*^ cells via Western blot analysis (Fig. 4E and Supplementary uncropped Western blots Fig. 4E). In addition, Mito-Tracker Red CMXRos staining showed that mitochondrial morphology was similar between the two groups in the control condition. After CaOX induction, however, mitochondria in *Acsl4*^*+/+*^ cells became fragmented and punctate, whereas those in *Acsl4*^*−/−*^ cells remained more intact and rod-shaped (Fig. 4F). JC-10 and BODIPY 581/591 C11 staining showed that, compared to *Acsl4*^*+/+*^ cells, *Acsl4*^*−/−*^ cells exhibited a higher proportion of red aggregates and a lower oxidized (Ox-C11) cell ratio, indicating a relatively higher mitochondrial membrane potential (Fig. 4G, J) and a lower level of lipid peroxidation (Fig. 4H, I) following CaOX treatment. Furthermore, using primary PTECs, we obtained similar findings (Fig. S6). Consistent with the in vivo experimental results, these observations implied that *Acsl4* knockout alleviated CaOX-induced mitochondrial dysfunction and reduced lipid peroxidation, thereby decreasing ferroptosis in MTECs.

### Upregulation of GPX4 decreased ferroptosis in MTECs following CaOX treatment

**Fig. 5:**
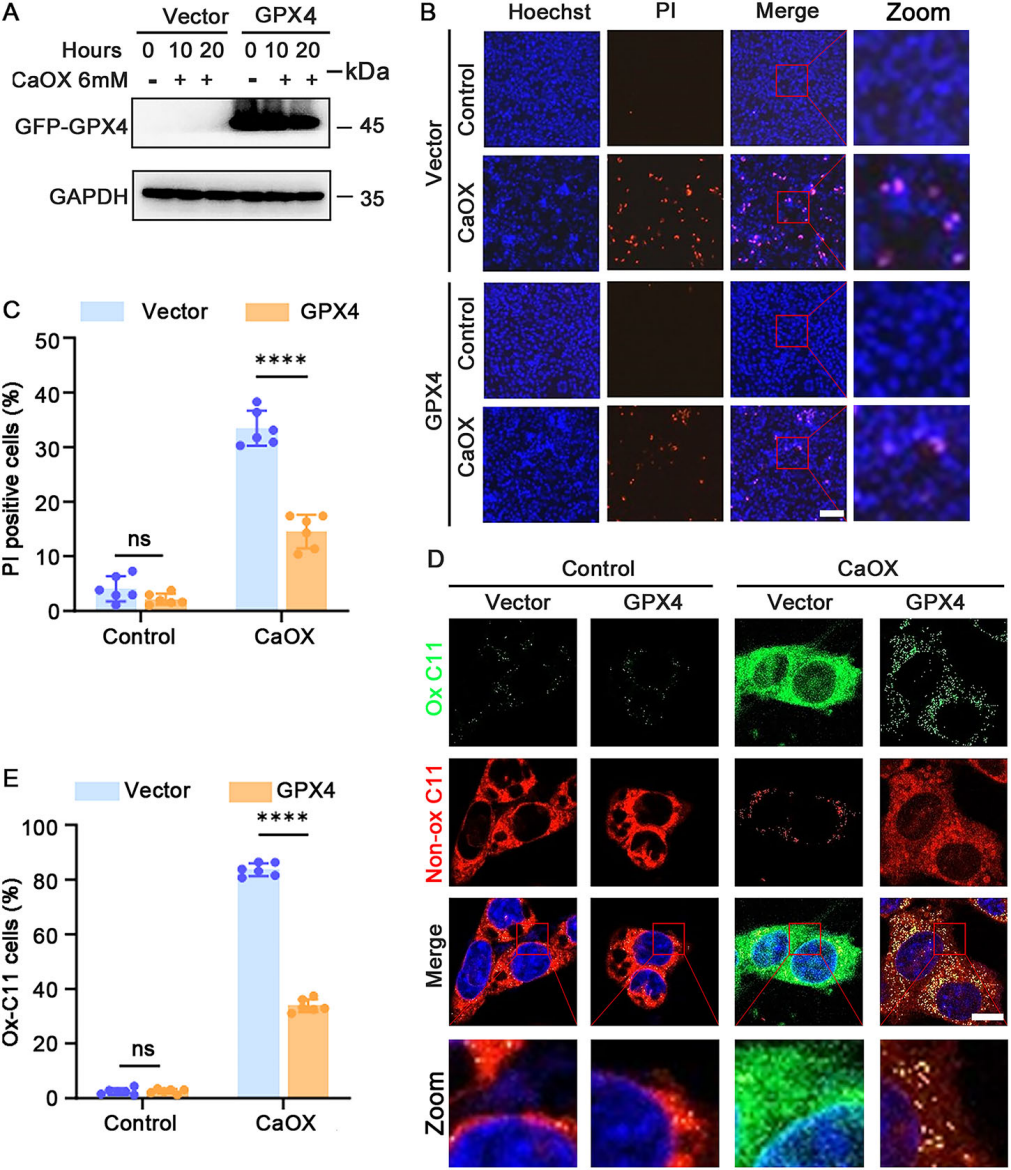
GPX4 overexpression decreased CaOX-induced ferroptosis in MTECs. **A** Western blot analysis of GFP-GPX4 (GPX4 protein with an N-terminal GFP tag) expression in MTECs during CaOX treatment. **B**, **C** Representative images of PI/Hoechst staining with quantification to assess cell death and **D**, **E** BODIPY 581/591 C11 staining with quantitative analysis to determine lipid peroxidation levels in MTECs treated with 6 mM CaOX (all *n* = 6, scale bar = 10 μm). ns nonsignificant, ****p < 0.0001.

To explore the influence of GPX4, a downstream regulator of the ACSL4 pathway, on ferroptosis in oxalate-induced AKI, we overexpressed GPX4 in MTECs. GPX4-overexpressing cells treated with CaOX exhibited stable GFP-GPX4 levels (Fig. 5A and Supplementary uncropped Western blots Fig. 5A), accompanied by a reduced PI-positive cell ratio (Fig. 5B, C) and decreased lipid peroxidation, as indicated by a lower Ox-C11 cell ratio (Fig. 5D, E). These findings indicated that GPX4 overexpression decreased CaOX-induced ferroptosis in MTECs.

### *Fsp1* knockout enhanced CaOX-induced ferroptosis in MTECs

**Fig. 6:**
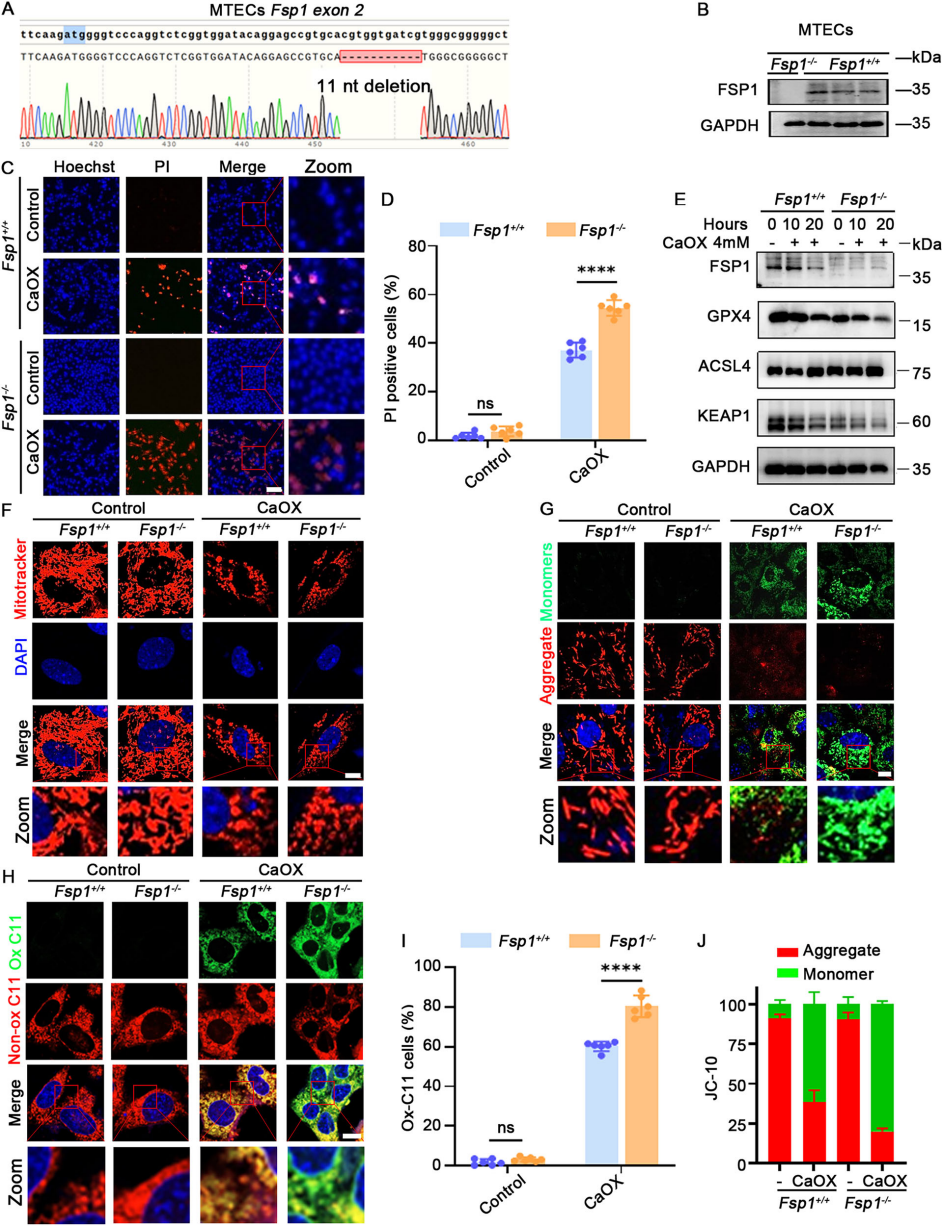
*Fsp1* knockout promoted CaOX-induced ferroptosis in MTECs. **A**, **B** Sanger sequencing and Western blot validation for *Fsp1* knockout in MTECs. **C**, **D** Representative images of PI/Hoechst staining and corresponding quantitative analysis in MTECs with 4 mM CaOX treatment for 20 h (*n* = 6, scale bar = 80 μm) and (**E**) the expression of ACSL4, FSP1, GPX4, and KEAP1 in MTECs following 4 mM CaOX treatment for 10 or 20 h. **F** Mito-Tracker Red CMXRos probe staining for examining the morphology of mitochondria, **G**, **J** representative images of JC-10 staining with quantification for the ratio of red aggregates and green monomers and **H**, **I** BODIPY 581/591 C11 probe staining for detecting ROS levels and quantitative analysis for the Ox-C11 cell ratios in MTECs with 4 mM CaOX treatment for 12 h (all *n* = 6, scale bar = 10 μm). ns nonsignificant, ****p < 0.0001.

To further investigate the role of *Fsp1* in oxalate-induced AKI, the CRISPR-Cas9 system was employed to create *Fsp1* knockout MTECs and confirmed through Sanger sequencing and Western blot analysis (Fig. 6A, B and Supplementary uncropped Western blots Fig. 6B). *Fsp1* knockout (*Fsp1*^−/−^) cells treated with CaOX exhibited a higher PI-positive ratio compared to *Fsp1* wild-type (*Fsp1*^+/+^) cells (Fig. 6C, D). Furthermore, Western blot analysis showed that *Fsp1*^−/−^ cells exhibited decreased expression of GPX4 and KEAP1, along with upregulated ACSL4 expression (Fig. 6E and Supplementary uncropped Western blots Fig. 6E). In addition, Mito-Tracker Red CMXRos staining revealed that, without CaOX treatment, mitochondrial morphology was similar between the two groups. However, following CaOX exposure, *Fsp1*^−/−^ cells exhibited extensive fragmentation, with mitochondria appearing as punctate structures (Fig. 6F). Moreover, JC-10 and BODIPY 581/591 C11 staining revealed that, compared to *Fsp1*^+/+^ cells, *Fsp1*^−/−^ cells showed a lower mitochondrial membrane potential (Fig. 6G, J) and a higher level of lipid peroxidation (Fig. 6H, I). Consistent results were also observed in primary PTECs (Fig. S7). In line with the in vivo experimental findings, these data demonstrated that *Fsp1* knockout aggravated CaOX-induced mitochondrial damage, intensified lipid peroxidation, and further enhanced ferroptosis in MTECs.

### FSP1 overexpression mitigated CaOX-induced ferroptosis in MTECs

**Fig. 7:**
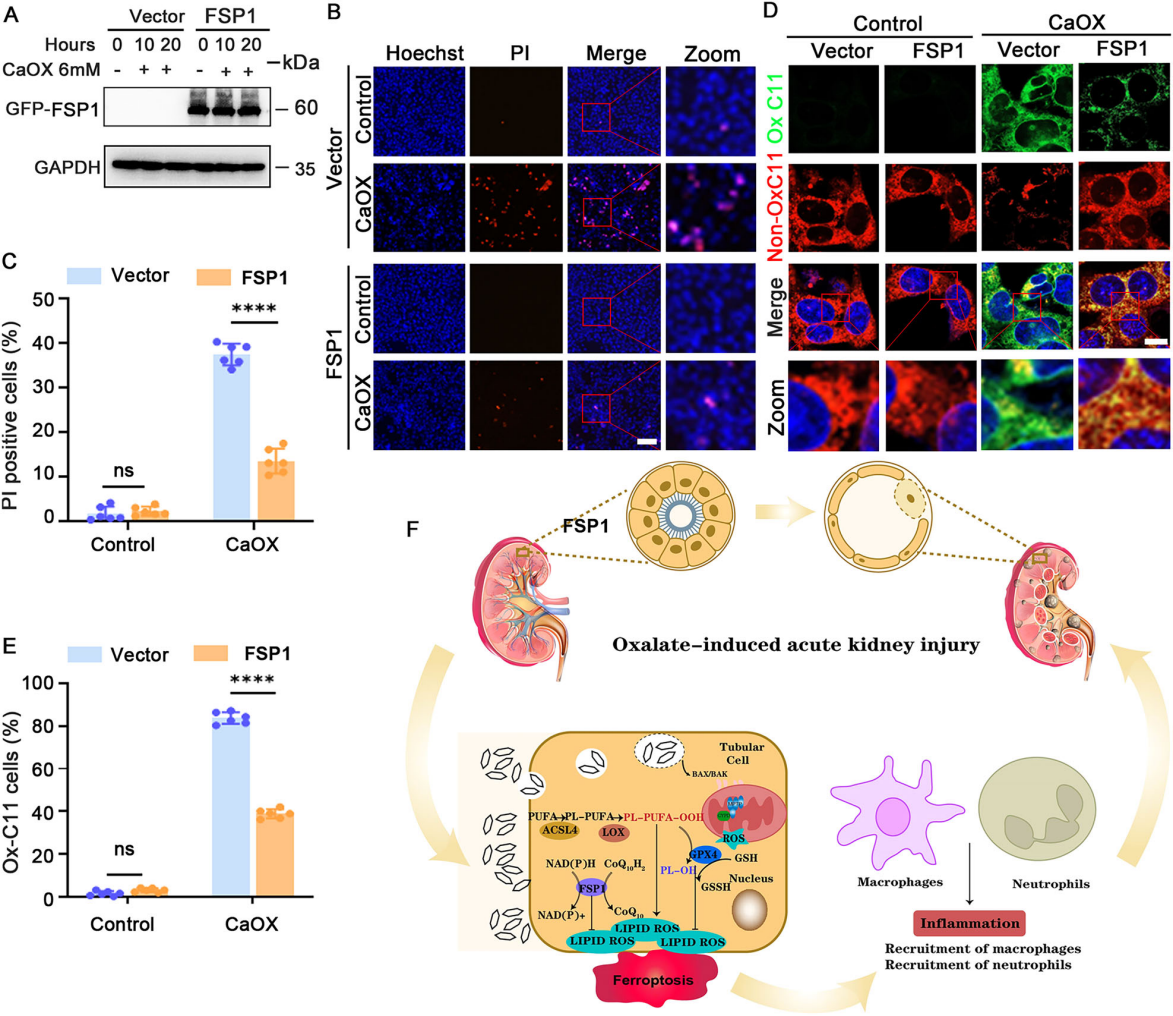
Upregulation of FSP1 reduced CaOX-induced ferroptosis in MTECs. **A** Western blot analysis for GFP-FSP1 (FSP1 protein with an N-terminal GFP tag) expression in MTECs during 6 mM CaOX treatment. **B**, **C** PI/Hoechst staining with quantification to assess cell death and **D**, **E** BODIPY 581/591 C11 staining with quantitative analysis to determine lipid peroxidation levels in MTECs treated with 6 mM CaOX (all *n* = 6, scale bar = 10 μm). ns, nonsignificant, ****p < 0.0001. **F** Schematic diagram: ferroptosis and the recruitment of macrophages and neutrophils in oxalate-induced AKI.

To elucidate the protective role of FSP1 in oxalate-induced AKI, we overexpressed FSP1 in MTECs. FSP1-overexpressing cells exhibited stable GFP-FSP1 levels following CaOX treatment (Fig. 7A and Supplementary uncropped Western blots Fig. 7A), along with a decreased ratio of PI-positive cells (Fig. 7B, C) and decreased lipid peroxidation, as indicated by BODIPY 581/591 C11 staining (Fig. 7D, E). These results suggested that FSP1 overexpression lessened ferroptosis in CaOX-treated MTECs.

## Discussion

Ferroptosis is an adaptive, programmed form of cell death that differs from apoptosis, driven by iron-mediated lipid oxidation within cell membranes [27, 28]. Suppressing ferroptosis has demonstrated the potential to slow the progression of various diseases, such as ischemia-reperfusion injury, ischemic cardiomyopathy, neurodegenerative diseases, organ transplant rejection, kidney damage and renal fibrosis [24]. Data from scRNA-seq and Western blot analyses of kidney tissues from mice with oxalate-induced AKI, along with observations of primary PTECs treated with CaOX, either alone or co-incubated with ferroptosis-specific inhibitors, demonstrated the occurrence of ferroptosis in renal tubular cells within the oxalate-induced AKI model. This study explored the roles of the two independent ferroptosis axes, ACSL4/GPX4 and FSP1, in oxalate-induced renal tubular epithelial cell injury, aiming to provide novel therapeutic approaches for treating oxalate-induced AKI.

ACSL4 regulates the metabolism of arachidonic acid and eicosapentaenoic acid, contributing to the induction of ferroptosis and driving lipid peroxidation in the pathogenesis of various diseases [29, 30]. Increased ACSL4 expression is observed in tumors, particularly triple-negative breast cancer, where higher ACSL4 levels may enhance sensitivity to radiotherapy and chemotherapy [31]. Studies in ischemia-reperfusion injury mouse models show that ACSL4 expression is upregulated in ischemic tissues, and inhibiting ACSL4 suppresses ferroptosis by reducing lipid peroxidation, significantly mitigating tissue and organ damage [32]. In this study, we found ACSL4 highly expressed in the kidney tissues of mice with oxalate-induced AKI. By knocking out *Acsl4* in renal tubules in vivo and in primary PTECs in vitro, we confirmed its critical role in oxalate-induced ferroptosis and kidney injury. ACSL4-driven ferroptosis involves the downstream molecule GPX4, which is essential for inhibiting ferroptosis by eliminating phospholipid hydroperoxides [33]. Disruption of the balance between these two factors is a determining factor in the onset of ferroptosis. The global deletion of *Gpx4* in mice causes early embryonic lethality, and its knockout in hepatocytes or neurons leads to perinatal or postnatal death [24]. Additionally, in our laboratory, *Gpx4*^*fl/fl*^*Ksp*^*Cre-ERT2*^ mice subjected to Tamoxifen induction (100 mg/kg) developed severe tubulointerstitial damage and acute tubular necrosis, accompanied by a marked impairment of renal function as early as day 5 post-induction (Fig. S8A–D). Furthermore, we employed the primary PTECs derived from non-4-hydroxytamoxifen (4-OHT)-induced *Gpx4*^*fl/fl*^*Ksp*^*Cre-ERT2*^ mice. Following 5 days of in vitro 4-OHT induction, near-total cellular death was observed, manifested by a significant elevation in PI-positive cells (Fig. S8E, F), preventing the successful generation of stable *Gpx4*-deficient PTECs. Therefore, we could only overexpress GPX4 in MTECs, which effectively reduced ferroptosis following CaOX treatment.

FSP1, another well-known suppressor of ferroptosis, acts independently of the ACSL4/GPX4 pathway. It reduces Coenzyme Q10 (CoQ10) to its antioxidant form, ubiquinol, which helps prevent lipid peroxidation [34]. It has been strongly linked to poor prognosis in malignant tumors [20], making it a promising therapeutic target for enhancing tumor cell ferroptosis. Additionally, increasing FSP1 expression to inhibit ferroptosis could facilitate nerve repair [35, 36] and offer a potential treatment strategy for Parkinson’s disease [37]. In this study, we observed reduced FSP1 expression in the kidneys of oxalate-induced AKI mice and found that its deficiency promoted ferroptosis, thereby exacerbating renal injury. Conversely, upregulation of FSP1 decreased ferroptosis, underscoring its protective role in oxalate-induced AKI.

Recent studies have highlighted the critical role of inflammation in stone disease models and in the kidney tissues of individuals with nephrolithiasis [38]. Macrophage-driven pro-inflammatory and anti-inflammatory responses play a critical role in the formation and progression of kidney stones [39, 40]. Besides macrophages, neutrophils are key inflammatory cells that contribute to the progression of AKI driven by various pathological factors [25, 41–43]. Our data demonstrated the pivotal role of ferroptosis in driving inflammatory infiltration in oxalate-induced AKI. ACSL4 deficiency diminished neutrophil and macrophage infiltration, while FSP1 deficiency exacerbated this process, suggesting their crucial involvement in the immune-inflammatory response during oxalate-induced AKI.

In summary, our study provided a comprehensive investigation of ferroptosis mediated by two independent axes, ACSL4/GPX4 and FSP1, in oxalate-induced AKI (Fig. 7F). Our findings indicated that targeting ACSL4 or, alternatively, upregulating GPX4 or FSP1 may help mitigate AKI by reducing ferroptosis, offering potential therapeutic strategies for oxalate-induced AKI.

## Methods

### Reagents and antibodies

Anti-Acsl4 antibody (ab155282, abcam), Anti-Fsp1 antibody (68049-1-Ig, Proteintech), Anti-Keap1 antibody (ab227828, Abcam), Anti-Gpx4 antibody (67763-1-Ig, Proteintech), Anti-F4/80 antibody (ab6640, Abcam), Anti- Ly6G antibody (ab25377, Abcam), Anti-Megalin antibody (ab184676, Aabcam), Anti-4-HNE antibody (ab48506, Abcam), Anti-GAPDH antibody (60004-1, Proteintech), BODIPY 581/591 C11 lipid peroxidation probe (D3861, Invitrogen), Mito-Tracker Red CMXRos fluorescent probe (C1035, Beyotime Biotechnology).

### Mice

All male mice used were on a C57BL/6J background. *Acsl4*^*fl/fl*^ mice were obtained from Cyagen Biosciences (Jiangsu), and *Fsp1*^*fl/fl*^ mice and *Ksp*^*Cre-ERT2*^ mice were sourced from GemPharmatech (Jiangsu). *Ksp*^*Cre*^ mice were generously provided by Professor Hui-Yao Lan from the Chinese University of Hong Kong. The *Acsl4* conditional knockout (cKO) model (*Acsl4*^*fl/fl*^*Ksp*^*Cre*^) was engineered through crossbreeding of *Acsl4*^*fl/fl*^ mice with *Ksp*^*Cre*^ transgenic strains, while the *Fsp1*^*fl/fl*^*Ksp*^*Cre*^ cKO mice were generated using analogous breeding strategies. *Acsl4*^*fl/fl*^*Ksp*^*Cre-ERT2*^ mice were generated by crossing *Acsl4*^*fl/fl*^ mice with *Ksp*^*Cre-ERT2*^ transgenic mice, and *Fsp1*^*fl/fl*^*Ksp*^*Cre-ERT2*^ mice were produced by crossing *Fsp1*^*fl/fl*^ mice with *Ksp*^*Cre-ERT2*^ mice. The genotype of the mice was confirmed using the Tail-Snip Polymerase PCR. *Acsl4*^*fl/fl*^*Ksp*^*Cre-ERT2*^ mice and *Fsp1*^*fl/fl*^*Ksp*^*Cre-ERT2*^ mice underwent tamoxifen-inducible recombination validation via PCR genotyping and subsequent Western blot confirmation of target protein ablation. A detailed description of their genetic background was provided in Fig. S2. All mice were housed and bred under specific pathogen-free conditions at the Laboratory Animal Center of Fujian Medical University, with a 12-h light/dark cycle and free access to food and water. Mice aged 10–12 weeks, weighing 25–28 g, were used for the experiments. All procedures adhered to Chinese regulations on animal care and use and were approved by the Animal Ethics Committee of Fujian Medical University.

### Oxalate-induced AKI model

Male mice were induced by a single intraperitoneal injection of 100 mg/kg (for *Acsl4*^*wt/wt*^*Ksp*^*Cre-ERT2*^ or *Acsl4*^*fl/fl*^*Ksp*^*Cre-ERT2*^ mice) or 60 mg/kg (for *Fsp1*^*wt/wt*^*Ksp*^*Cre-ERT2*^ or *Fsp1*^*fl/fl*^*Ksp*^*Cre-ERT2*^ mice) NaOX and were fed 3% NaOX in drinking water for 1 or 2 days. Our preliminary results indicated that *Fsp1* knockout mice exhibited increased susceptibility to oxalate-induced AKI. Therefore, we administered a lower dose (60 mg/kg) of NaOX to *Fsp1*^*wt/wt*^*Ksp*^*Cre-ERT2*^ and *Fsp1*^*fl/fl*^*Ksp*^*Cre-ERT2*^ mice. The investigators were blinded to the outcome assessment. Kidney samples were collected on days 0, 1, and 2. Hematoxylin and eosin (HE) staining and Von-Kossa staining were used to confirm the successful establishment of the model.

### Isolation and culture of primary proximal tubule epithelial cells (PTECs)

Fresh mouse renal cortex was minced on ice and digested in HBSS with 0.75 mg/mL trypsin inhibitor and 0.75 mg/mL collagenase at 37 °C for 20 min. Digestion was halted with DMEM/F12 containing 10% FBS on ice. The cell suspension was filtered through a 100-μm sieve and centrifuged at 2000×*g* with 33% Percoll. After washing with HBSS to remove Percoll, cells were resuspended in DMEM/F12 with 10% FBS and plated on collagen-coated plates and cultured at 37 °C in a 5% CO_2_ incubator.

### Generation of FSP1 and GPX4 overexpressing MTECs

HEK293T cells were transfected with packaging plasmids and pBOBI-C-GFP-mFsp1 or pBOBI-N-GFP-mGpx4 vectors using calcium phosphate. After 12 h, the medium was replaced, and cells were cultured for another 48 h. Virus-containing supernatant was collected by centrifugation at 2500 rpm for 3 min. MTECs at 50–70% confluence were transfected with the virus (MOI 100) in the presence of 8–10 μg/mL polybrene. After centrifuging at 2500 rpm for 30 min at 37 °C, cells were returned to the incubator. Infection efficiency was checked by Western blot after 48 h.

### Generation of *Acsl4* and *Fsp1* gene knockout cells using the CRISPR-Cas9 system

The pBOBI cas9+gRNA puro shuttle lentiviral plasmid was packaged into viruses to infect MTECs with 10 μg/mL polybrene. Cas9, guided by sgRNAs targeting *Acsl4* (5′-TGAAATGCAGCCAAATGGAA-3′) or *Fsp1* (5′-TGCACGTGGTGATCGTGGGC-3′) [44], mediated cleavage of these genes. Cells were selected with 2 μg/mL puromycin, and monoclonal cells were obtained using the limiting dilution method and confirmed by sequencing and Western blot analysis.

### Western blot

Protein was extracted from renal tissues or MTEC cells, and concentrations were measured using a BCA protein assay kit. Samples were boiled for 5 min and loaded onto SDS-PAGE gels for electrophoresis at 80 V. Proteins were transferred to PVDF membranes and blocked with 5% non-fat milk at room temperature for 1 h. The membranes were incubated overnight at 4 °C with primary antibodies: ACSL4, FSP1, KEAP1, and GPX4 (1:1000), and GAPDH (1:5000). After three washes with TBST, membranes were incubated with goat anti-rabbit or mouse secondary antibody (1:5000) at room temperature for 1 h, followed by three 10-min washes. Protein bands were detected using an ECL kit and chemiluminescence imaging, and band intensities were analyzed with Image J.

### Statistical analysis

The results are presented from at least three independent experiments. Statistical analyses and data visualization were conducted using GraphPad Prism 9.5 (GraphPad Software, USA). The normality of all quantitative variables was confirmed using the Shapiro–Wilk test, and the data were consequently expressed as mean ± standard deviation (SD). The homogeneity of variances was verified using the *F*-test for comparisons between two groups and Bartlett’s test for comparisons among three or more groups. To evaluate statistically significant differences, an unpaired Student’s *t*-test was employed for comparisons between two groups, while one-way ANOVA was utilized for comparisons among three or more groups. Multiple comparisons with Bonferroni’s correction were applied following ANOVA. All statistical tests were two-tailed, and a *p* value <0.05 was considered statistically significant.

## Supplementary information

Supplementary Information

Original Western blots

## Data Availability

All data necessary to assess the conclusions of this paper are included within the manuscript. Further data related to this study can be obtained by contacting the corresponding author.
