# Supplementary material for: Roles of ACSL4/GPX4 and FSP1 in oxalate-induced acute kidney injury
Source: Cell Death Discov. 2025 Jun 17;11:279. doi: 10.1038/s41420-025-02557-y (PMC12174353; doi:10.1038/s41420-025-02557-y)
Supplement: Supplementary file 1 — Supplementary Information [file 41420_2025_2557_MOESM1_ESM.pdf]

Supplementary Methods, Figures and Figure Legends

Supplementary Methods

JC-10 assay for mitochondrial membrane potential

Cells were seeded in 35 mm dishes and treated with 6 mM or 4 mM CaOX for 12 hours. After washing with serum-free medium, cells were incubated with JC-10 at 37°C for 30 minutes. Cells were washed twice and imaged by fluorescence microscopy. High mitochondrial membrane potential causes JC-10 to form red-fluorescent aggregates, while low potential results in green-fluorescent monomers. Mitochondrial membrane potential changes, reflected by the fluorescence color shift, indicate the integrity of the mitochondrial membrane.

BODIPY 581/591 C11 probe for lipid oxidation detection

BODIPY 581/591 C11 is emitted at 591 nm (reduced prototype), or red-shifted to 510 nm (oxidized type). Briefly, cells are treated with 6 mM or 4 mM CaOX for 12 hours, then washed and incubated with 10 μM C11 BODIPY 581/591 at 37°C for 30 minutes. After three PBS washes, the 590 nm/510 nm fluorescence ratio is measured by confocal microscopy to reflect the lipid peroxidation level.

Mito-Tracker Red staining for mitochondrial morphology assessment

After 12 hours of 6 mM or 4 mM CaOX treatment, cells are incubated with 200 nM Mito-Tracker Red at 37ºC for 15 minutes. After washing with serum-free medium, cells are ready for confocal microscopy imaging.

Immunofluorescence staining

Kidney cryosections were equilibrated at room temperature for 30 minutes, then fixed with ice-cold acetone at 4°C for 10 minutes. After three PBS washes, sections were blocked with 10% goat serum for 30 minutes. Primary antibodies were diluted as per instructions and incubated overnight at 4°C. After washing with PBS, sections were incubated with fluorescent secondary antibodies (1:1000) for 1 hour at room temperature. Following DAPI staining for 10 minutes, slides were mounted with anti-fade medium and imaged under a fluorescence microscope.

PAS Staining

4 μm thick mouse kidney tissue sections were deparaffinized, rehydrated, and treated with periodic acid for 10 minutes, then stained with Schiff's reagent for 10 minutes. After a 5-minute rinse, nuclei were stained with hematoxylin for 3 minutes. Sections were then dehydrated, cleared, and mounted. Tissue injury was evaluated under a light microscope. Tubular injury was scored in 6 randomly selected high-power fields per sample using the following criteria: 0 = no injury, 1 = mild injury (epithelial swelling, tubular dilation), 2 = severe injury (flattened epithelium, nuclear loss, tubular obstruction), and 3 = extensive damage (epithelial disruption, cell detachment, nuclear loss, casts). Scores were averaged from 6 fields in a double-blind randomized analysis.

HE staining

Paraffin sections (4 μm thick) were deparaffinized, rehydrated, and rinsed with distilled water. Hematoxylin staining was performed for 3-8 minutes, followed by tap water rinsing and brief differentiation with 1% acid alcohol. After washing, 0.6%

ammonia water was applied to blue the stain. Eosin staining followed for 1-3 minutes. The sections were then dehydrated, cleared, and mounted for evaluation of tissue injury under a light microscope.

Von-Kossa staining

4 μm thick paraffin sections were baked at 65°C for 2 hours, deparaffinized with xylene, and rehydrated through a graded alcohol series. Sections were treated with Von Kossa silver solution under strong light for 10-60 minutes, rinsed with distilled water, and immersed in hypo solution for 2 minutes. Nuclear staining was performed with hematoxylin and counterstained with neutral red. Finally, sections were dehydrated, cleared, and mounted.

Supplementary Figures and Figure Legends

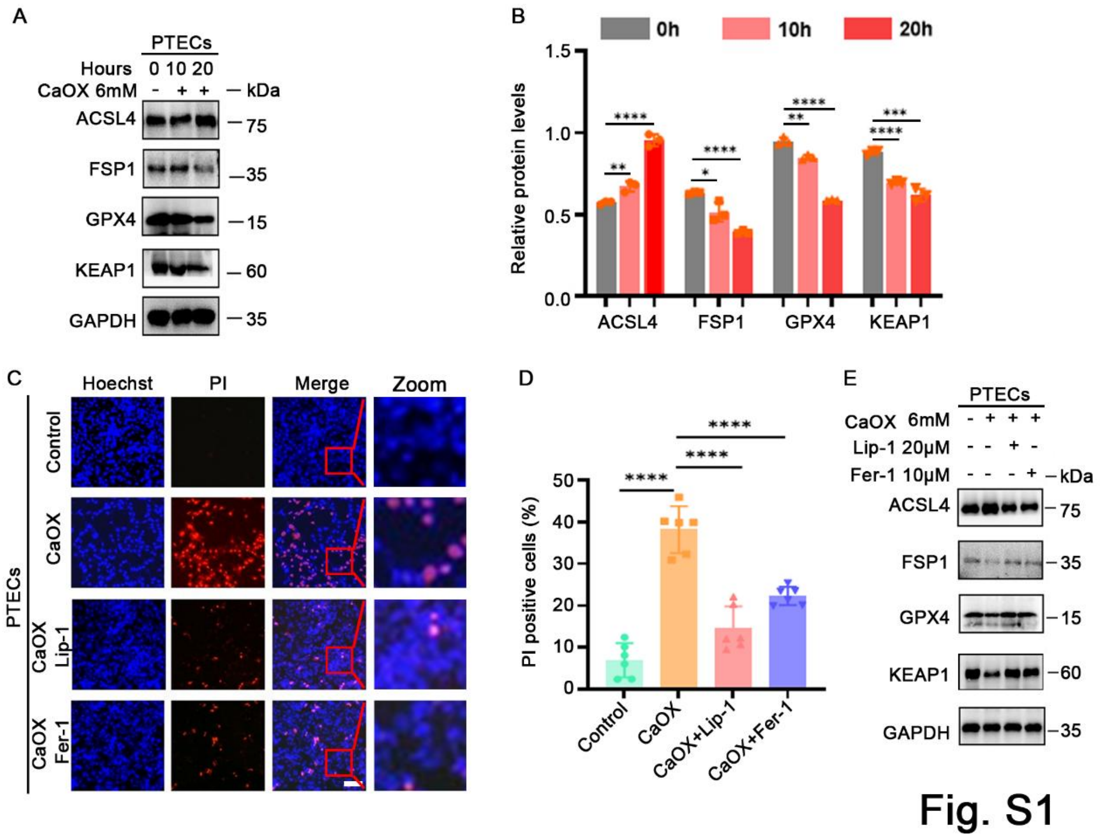

Fig. S1 CaOX altered the expression of ferroptosis-related proteins in PTECs and ferroptosis inhibitors protected against CaOX-induced cell death.

(A-B) Western blot analysis of ACSL4, FSP1, GPX4, and KEAP1 expression in PTECs following 6 mM CaOX treatment (n = 3). (C-D) Representative images of PI/Hoechst staining (n = 6, scale bar = 80 μm) and (E) Western blot analysis of ferroptosis-related protein expression in PTECs co-treated with 6 mM CaOX and the ferroptosis inhibitors Lip-1 (20 μM) or Fer-1 (10 μM). *$p < 0.05$, **$p < 0.01$, ***$p < 0.001$, ****$p < 0.0001$

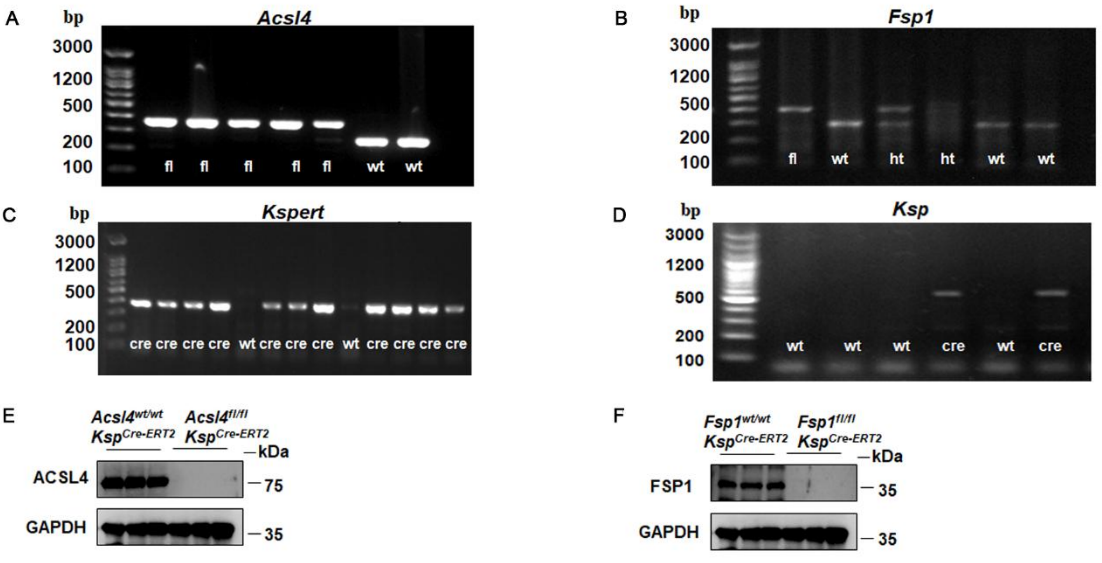

Fig. S2

Fig. S2 Specific deletion of *Acsl4* and *Fsp1* in renal tubules.

(A-D) Genotyping of *Acsl4* and *Fsp1* by tail-snip PCR amplification. Representative DNA gel electrophoresis results of *Acsl4*$^{wt/wt}$, *Acsl4*$^{fl/fl}$ mice and heterozygotes. One DNA band at about 350 bp is observed for *Acsl4*$^{fl/fl}$ mice, while the one at about 220 bp is observed for wild-type (WT)pups. If both bands are shown, indicating heterozygotes. (B) Representative DNA gel electrophoresis results of *Fsp1*$^{wt/wt}$, *Fsp1*$^{fl/fl}$ mice and heterozygotes. One DNA band at about 396 bp is observed for *Fsp1*$^{fl/fl}$ mice, while the one at about 275 bp is observed for WT pups. If both bands are shown, indicating for heterozygotes. (C) Representative DNA gel electrophoresis results of *Ksp*$^{Cre\text{-}ERT2}$. DNA band at about 355 bp is observed for *Ksp*$^{Cre\text{-}ERT2}$ mice. (D) Representative DNA gel electrophoresis results of *Ksp*^{Cre}. DNA band at about 420 bp is observed for *Ksp*^{Cre} mice. (E-F) Western blot analysis for ACSL4 and FSP1 in WT and conditional knockout (cKO) mice.

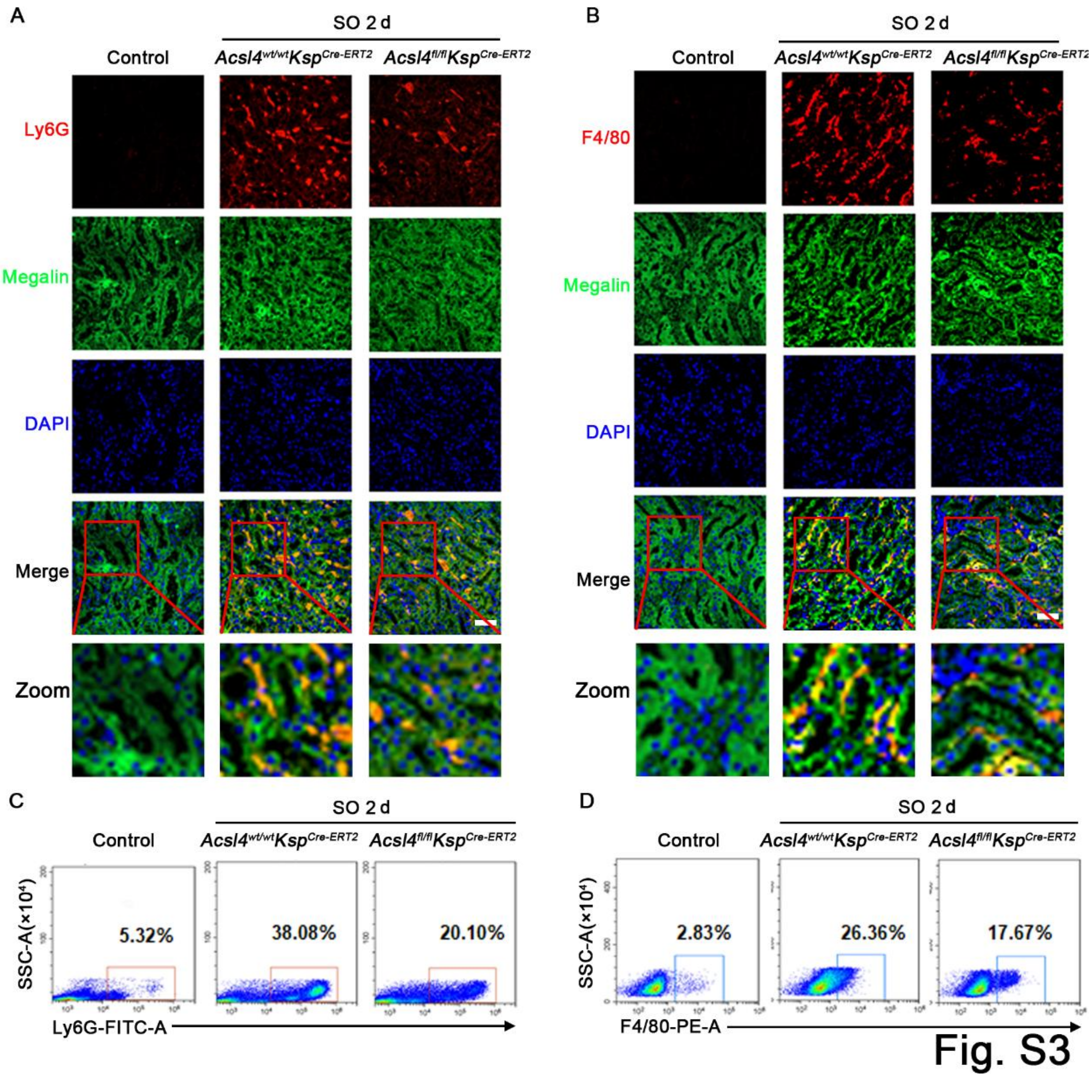

Fig. S3 ***Acsl4*** **deletion reduced renal inflammatory cell infiltration in oxalate-induced AKI.**

(A-B) Immunofluorescence staining for infiltration of neutrophils and macrophages in renal tissues of oxalate-induced AKI. Ly6G (neutrophils, red), F4/80 (macrophages, red), Megalin (renal tubules, green), DAPI (nuclei, blue), the control group consists of $Acsl4^{wt/wt}Ksp^{Cre\text{-}ERT2}$ mice without SO administration, n = 6, scale bar = 50 μm. (C-D) Flow cytometry showing the percentage of neutrophils (Ly6G^{+}, gated from CD45^{+}) and macrophages (F4/80^{+}, gated from CD45^{+}CD11b^{+}) infiltrating the kidneys in oxalate-induced AKI (n = 3).

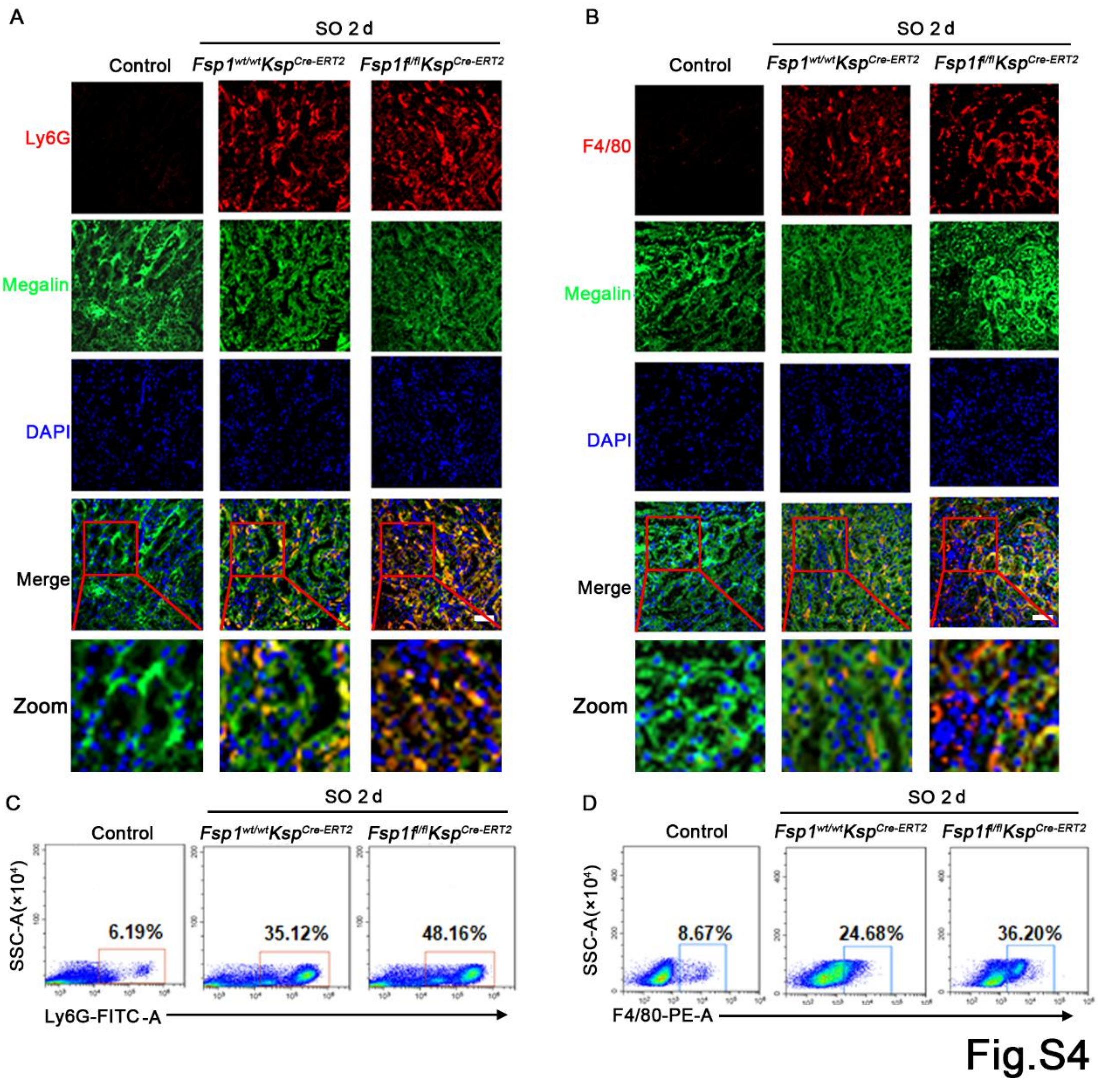

Fig. S4 *Fsp1* deletion promoted renal inflammatory cell infiltration in oxalate-induced AKI.

(A-B) Immunofluorescence staining, the control group consists of $Acsl4^{wt/wt}Ksp^{Cre\text{-}ERT2}$ mice without SO administration (n = 6, scale bar = 50 μm). (C-D) Flow cytometry for neutrophils ($Ly6G^{+}$) and macrophages ($F4/80^{+}$) infiltration in renal tissues of oxalate-induced AKI (n = 3).

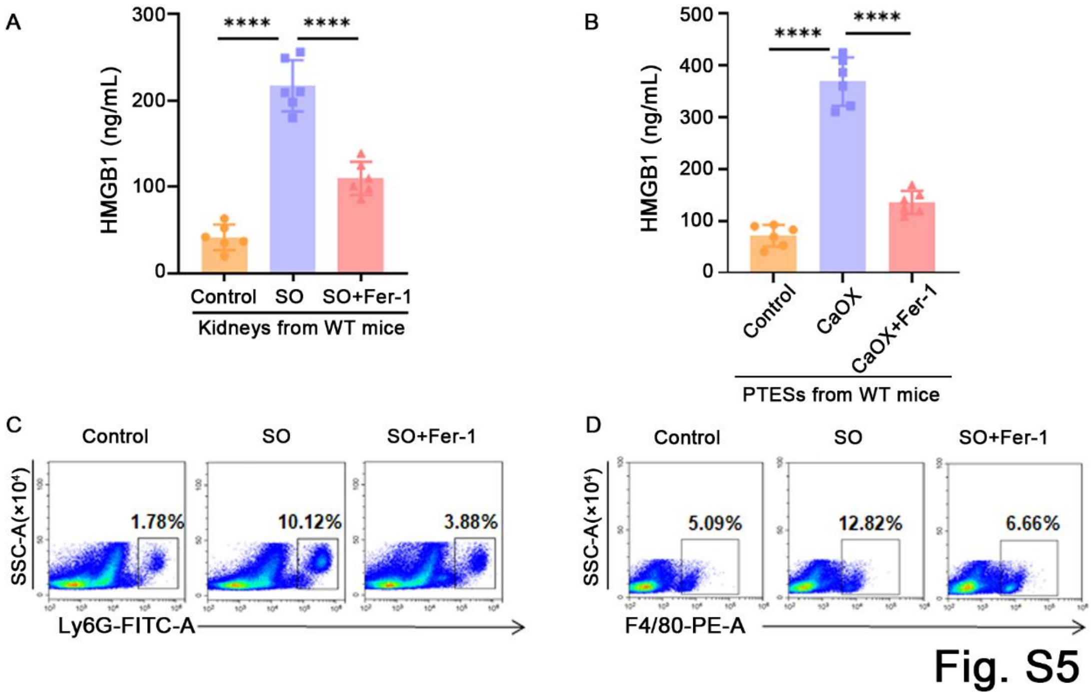

Fig. S5 Fer-1 inhibited the release of HMGB1 and renal inflammatory cell infiltration in oxalate-induced AKI.

(A-B) Quantitative analysis of HMGB1 levels in kidneys and PTECs from WT mice assessed by ELISA (n = 6). (C-D) Flow cytometry for neutrophils (Ly6G$^+$) and macrophages (F4/80$^+$) infiltration in renal tissues of oxalate-induced AKI mice after Fer-1 administration (n = 6). ****$p < 0.0001$

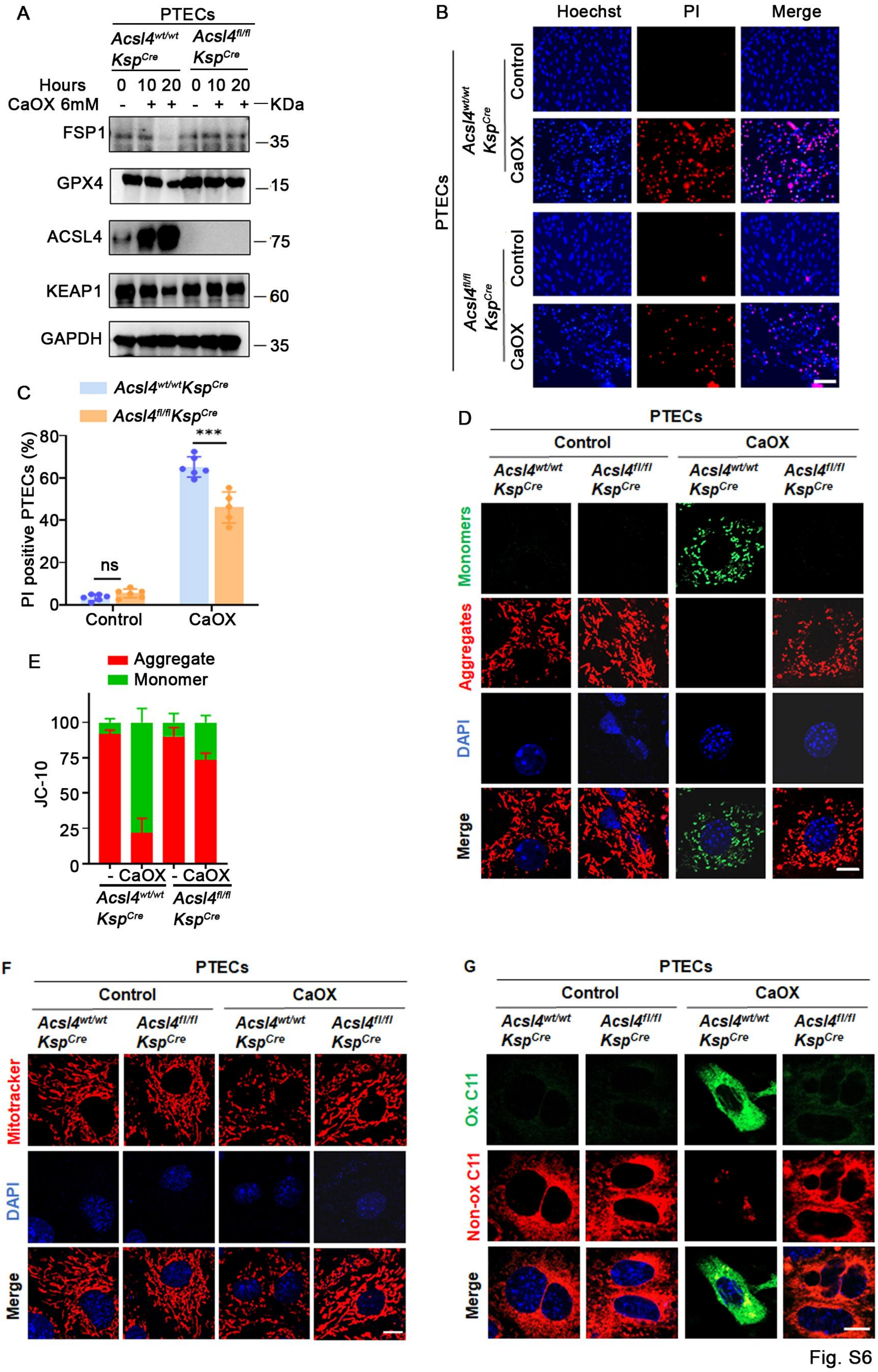

Fig. S6 Deletion of *Acsl4* alleviated ferroptosis in PTECs.

(A-B) Representative images of PI/Hoechst staining and corresponding quantitative analysis in PTECs with 6 mM CaOX treatment for 20 hours (n = 6, scale bar = 80 μm) and (C) the expression of ACSL4, FSP1, GPX4, KEAP1 in PTECs after 6 mM CaOX treatment for 10 or 20 hours. (D-E) JC-10 staining for assessing mitochondrial membrane potential with quantitative analysis of the ratio between red aggregates and green monomers, (F) Mito-Tracker Red CMXRos probe staining for examining the morphology of mitochondria, and (G) representative images of BODIPY 581/591 C11 lipid peroxidation fluorescent probe staining for the oxidized (Ox-C11) cell ratios in PTECs with 6 mM CaOX treatment for 20 hours (all n = 6, scale bar = 10 μm). ns, nonsignificant, $***p < 0.001$

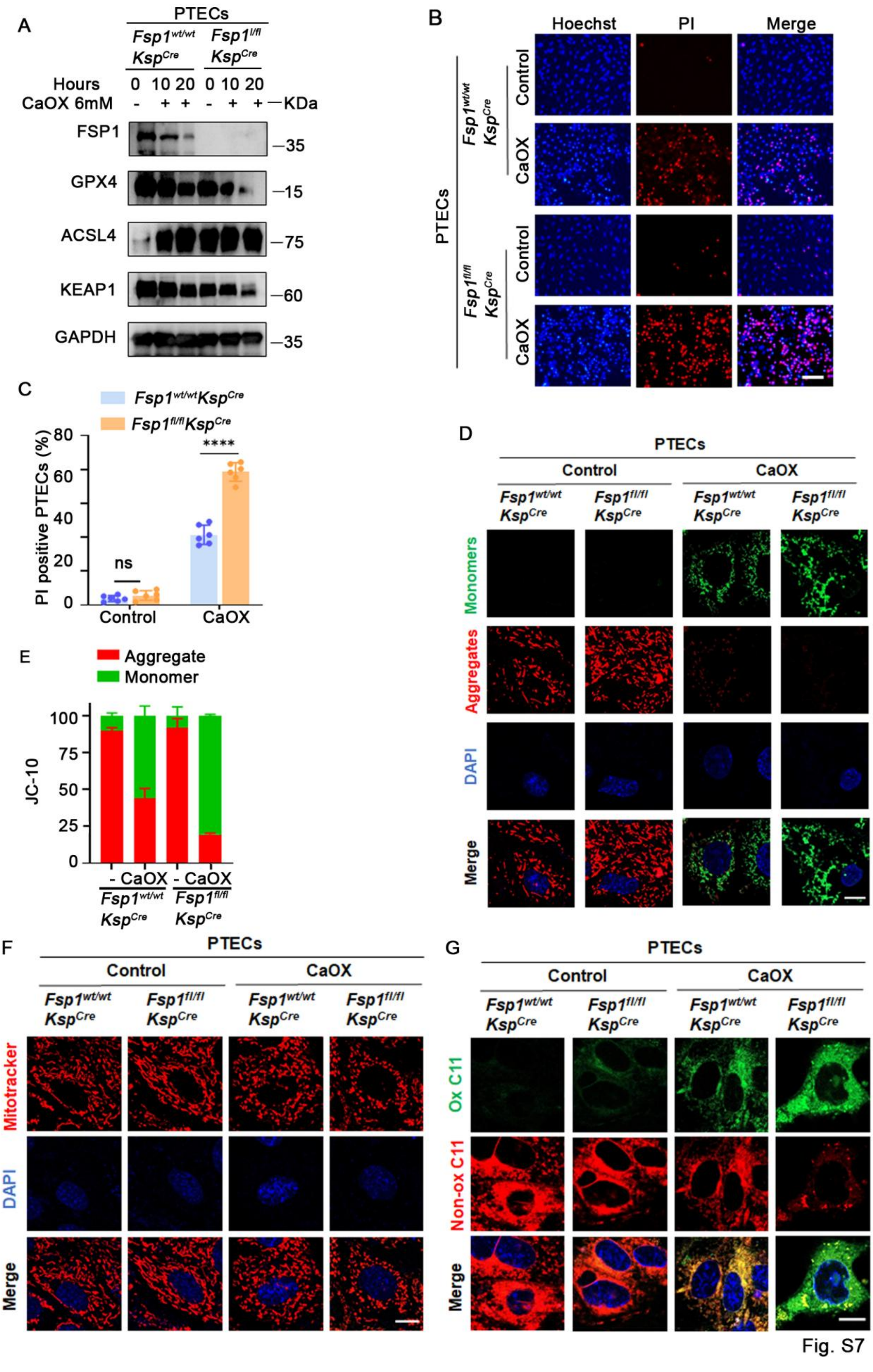

Fig. S7 Deletion of *Fsp1* exacerbated ferroptosis in PTECs.

(A-B) Representative images of PI/Hoechst staining and corresponding quantitative analysis in PTECs with 4 mM CaOX treatment for 20 hours (n = 6, scale bar = 80 μm) and (C) the expression of ACSL4, FSP1, GPX4 and KEAP1 in PTECs following 4 mM CaOX treatment for 10 or 20 hours. (D-E) Representative images of JC-10 staining with quantification for the ratio of red aggregates and green monomers, (F) Mito-Tracker Red CMXRos probe staining for examining the morphology of mitochondria and (G) BODIPY 581/591 C11 probe staining for detecting ROS levels for the Ox-C11 cell ratios in PTECs with 4 mM CaOX treatment for 20 hours (all n = 6, scale bar = 10 μm). ns, nonsignificant, ****$p < 0.0001$

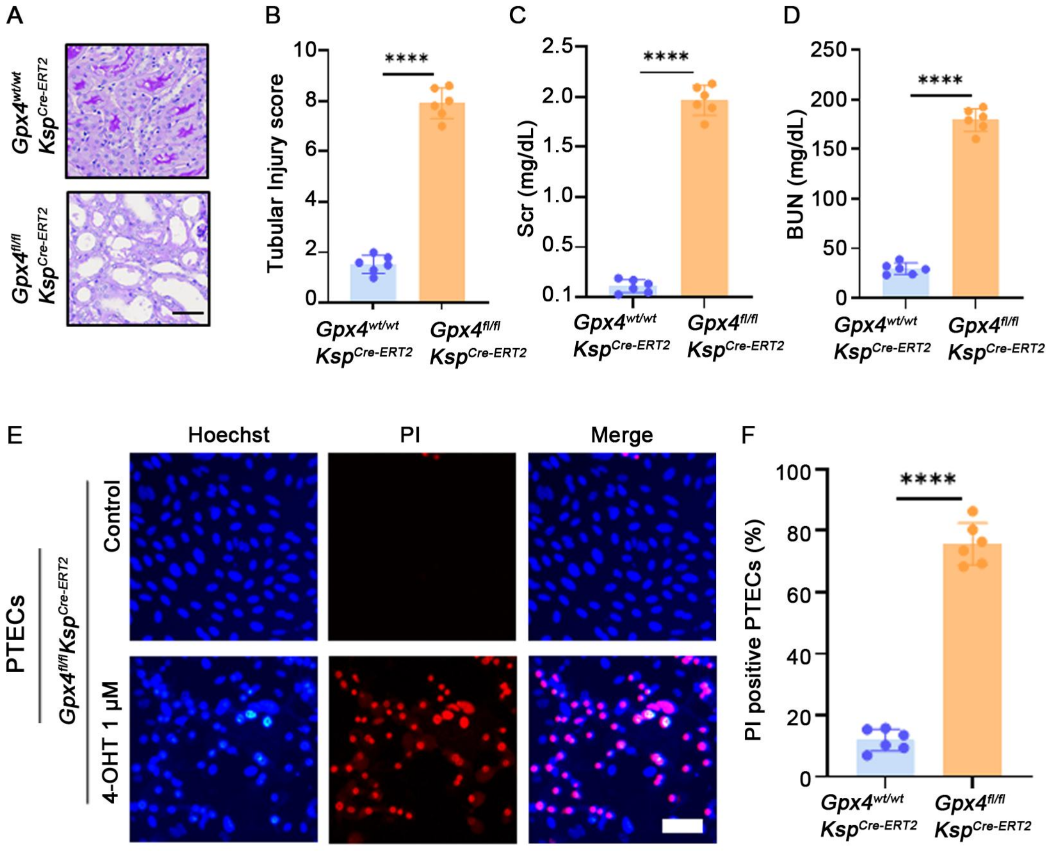

Fig. S8

Fig. S8 Conditional knockout of *Gpx4* in renal tubules led to significant dysfunction and cell death.

(A) PAS staining of kidney sections and (B) tubular injury score in the indicated mice (n = 6). (C-D) Evaluation of kidney function, including the measurement of Scr and BUN levels (n = 6). (E-F) Representative images of PI/Hoechst staining and corresponding quantitative analysis in 4-OHT-induced $Gpx4^{fl/fl}Ksp^{Cre\text{-}ERT2}$ PTECs (n = 6, scale bar = 40 μm). ****$p < 0.0001$.
