# Supplementary figures and images for: Roles of ACSL4/GPX4 and FSP1 in oxalate-induced acute kidney injury
Source: Cell Death Discov. 2025 Jun 17;11:279. doi: 10.1038/s41420-025-02557-y (PMC12174353; doi:10.1038/s41420-025-02557-y)

Fig1D

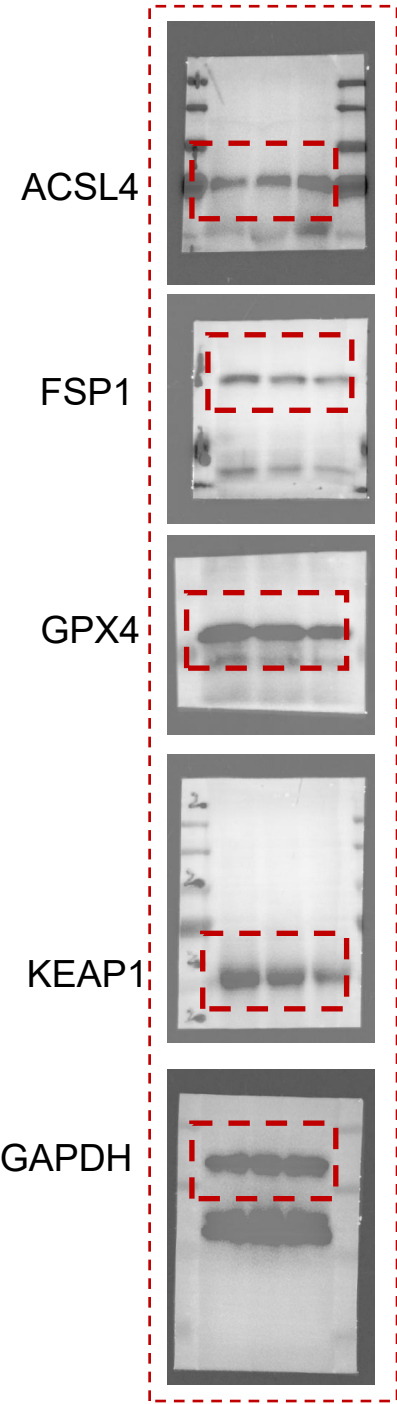

Fig4B

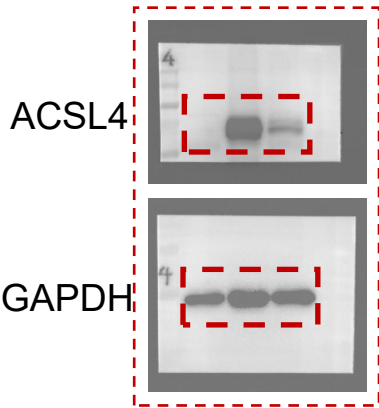

Fig4E

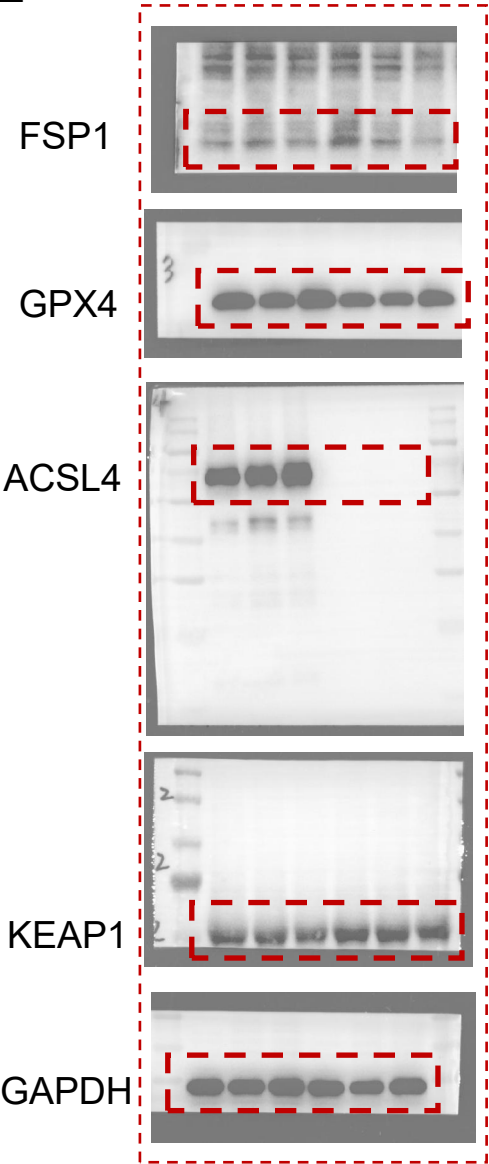

Fig5A

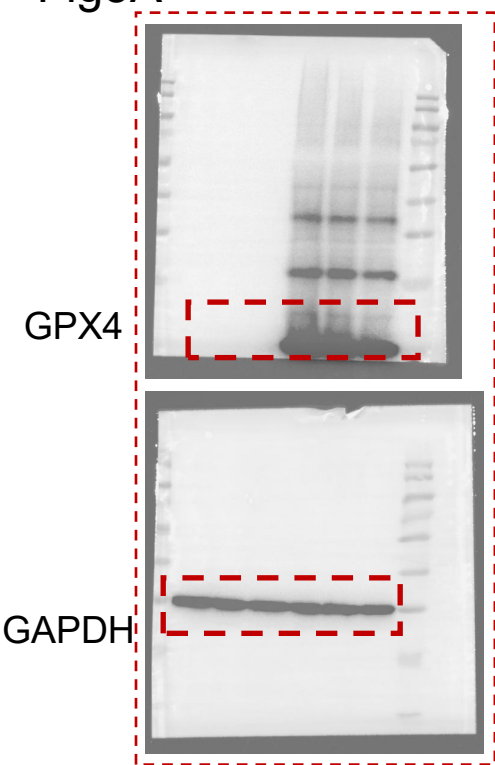

Fig6B

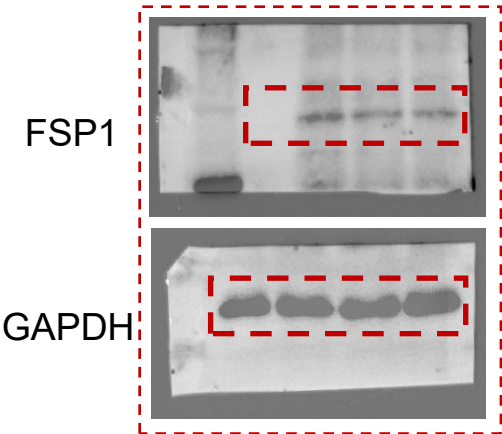

Fig6E

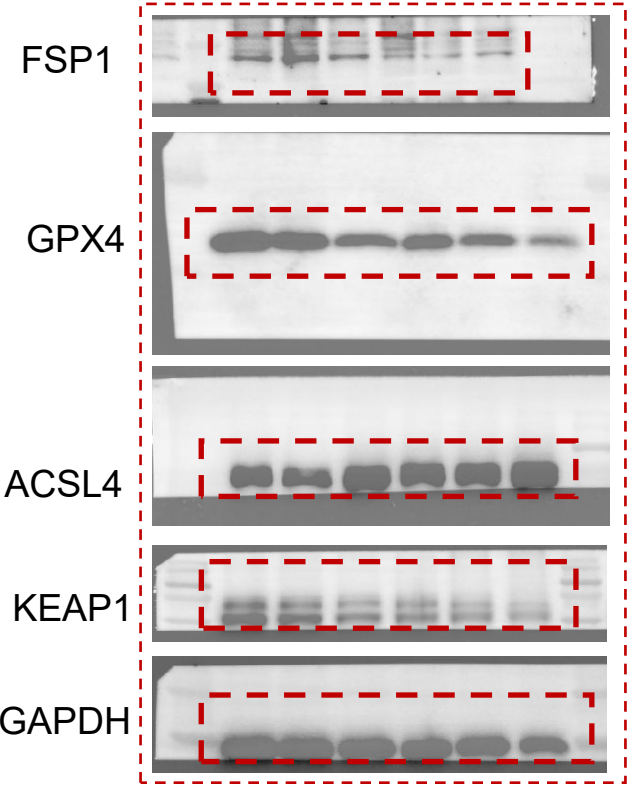

Fig7A

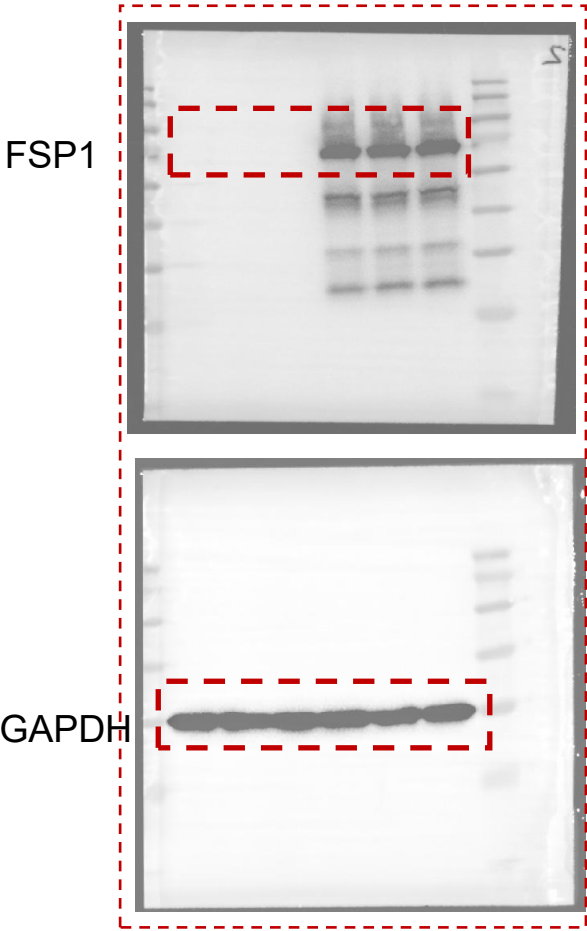

FigS1A

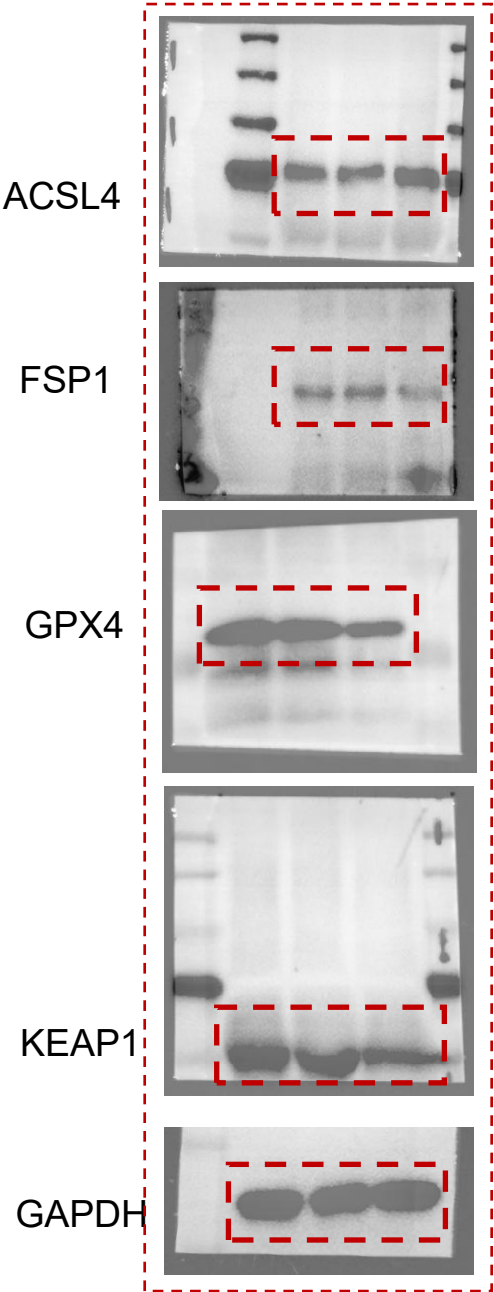

FigS1E

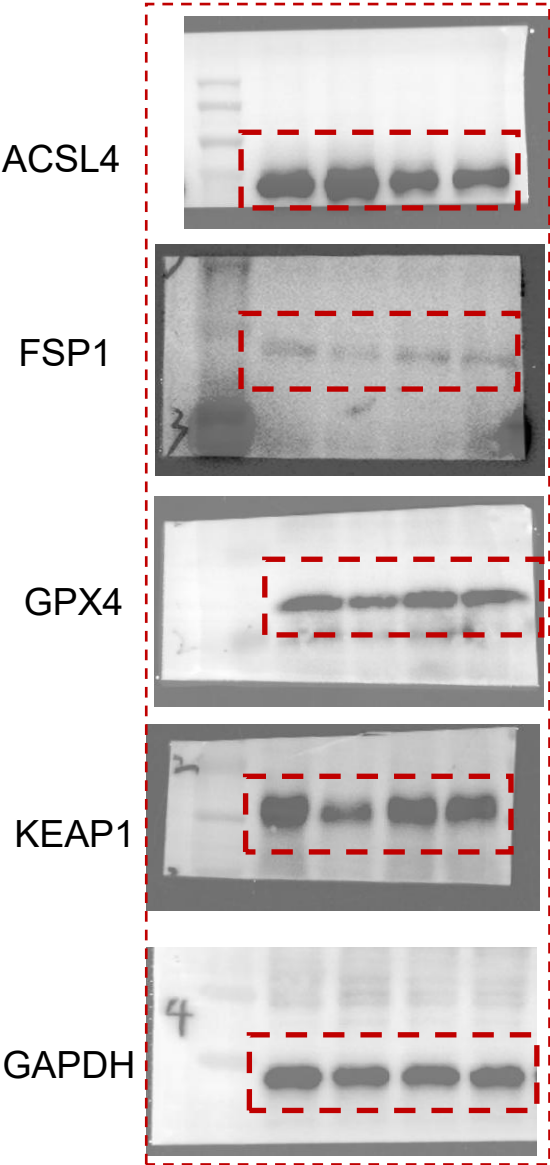

Supplement: Supplementary file 2 — Original Western blots [file 41420_2025_2557_MOESM2_ESM.pdf]
